# Easily Accessible and Up‐Scalable Aliphatic Bis‐Formamides with Afterglow Luminescence: Photoluminescence Properties and Applications

**DOI:** 10.1002/smll.74099

**Published:** 2026-06-16

**Authors:** Chengyi Zhu, Sara Ferrara, Anna Zieleniewska, Daniel Van Opdenbosch, Rubén D. Costa, Youssef Atoini, Jean‐Marie Lehn

**Affiliations:** ^1^ Lehn Institute of Functional Materials School of Chemistry Sun Yat‐Sen University Guangzhou China; ^2^ Laboratoire de Chimie Supramoléculaire Institut De Science et d'Ingénierie Supramoléculaires University of Strasbourg Strasbourg France; ^3^ Campus Straubing For Biotechnology and Sustainability Chair of Biogenic Functional Materials Technical University of Munich Straubing Germany; ^4^ Campus Straubing For Biotechnology and Sustainability Chair for Biogenic Polymers Technical University of Munich Straubing Germany

**Keywords:** afterglow, aggregation, bis‐formamides, emission recovery, white hybrid light‐emitting diodes

## Abstract

Materials based on water‐processable, non‐conjugated organic molecules featuring afterglow luminescence are promising due to their sustainable character and their straightforward upscale. Yet, they remain barely applied in advanced optoelectronics, such as solid‐state lighting and anti‐counterfeiting, because of a lack of mechanistic studies. Herein, we describe a class of terminal bis‐formamide compounds, H(O═)CNH─(CH_2_)_n–_HNC(═O)H, that feature a long afterglow with an average excited‐state lifetime (<τ>) close to the second. As an interesting archetype, N,N'‐(hexane‐1,6‐diyl) bis‐formamide (*n* = 6, **BFAC6**) exhibits a <τ> of 0.40 s associated with a photoluminescence quantum yield of 13% in both crystalline solid state and polyvinyl alcohol films (**BFAC6@PVA**). Thorough investigations such as temperature‐dependent x‐ray analyses, time‐resolved electronic spectroscopy, and electrochemical impedance spectroscopy suggest that the unusual long‐lived emission could be attributed to rigid packing ruled by H‐bonding, resulting in an emitting excited state attributed to— at least— a H‐trapped emissive state. Moreover, the so‐called clustering‐triggered emission, or clusteroluminescence, is also to be considered here. As a proof of concept, these materials were used for decoration, anti‐counterfeiting, and color down‐conversion for white hybrid light‐emitting diodes, featuring promising performances in terms of device stability, as well as recovery of the initial emission features upon heating–cooling processes.

## Graphical abstract

A bis formamide molecule was found to feature afterglow properties, in both crystalline powder state and in polyvinyl alcohol films. This was attributed to the intermolecular packing that allows H‐trapped emitting state and/or clustering‐triggered emission, which was intensified upon insertion of tetramethylbenzidine as a doping agent. This material was used in several applications such as decoration, anti‐counterfeiting, and white hybrid LEDs.

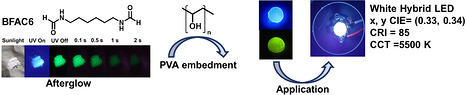

## 1 Introduction

Molecules and materials featuring the afterglow luminescence phenomenon are of high interest owing to their promising applications in multiple fields, such as optoelectronics [1, 2], bioimaging [3], chemo‐ and biosensors [4, 5], and anti‐counterfeiting technology [6], as well as its importance in unraveling the fascinating dynamics of excitons in organic materials [7, 8]. High photoluminescence quantum yields (φ) and long excited‐state lifetimes (<τ>) are the main parameters for evaluating afterglow processes [9]. In this context, numerous strategies, such as crystallization [10, 11, 12], host‐guest doping [13, 14], heavy‐atom effect [9, 15], organic–inorganic hybridation [6, 16] and many others [17, 18, 19], have been brought forward to develop ideal afterglow materials [20].

Among them, most of the reported organic luminescent materials are based on π‐extended molecules [21, 22, 23] that require demanding synthesis and high costs, making large‐scaling hardly feasible. Hence, the research community has dedicated efforts on more easily available, non‐conjugated organic compounds featuring afterglow properties [24, 25, 26, 27, 28, 29]. In a nutshell, design rules for promoting and/or enhancing afterglow involve crystallization [30, 31], halogen bonding [15], H‐aggregation [32, 33], host‐guest, n‐π* transitions [34], dopant‐based systems [35], and ionic bonding [36]. As example, two non‐aromatic succinimide compounds with effective through‐space intermolecular interactions result in diverse emissive clusters with long persistent emission [28]. Here, strong intermolecular interactions along with n → π* orbital overlap were proposed as the key features underlying afterglow luminescence triggered by cluster formation. Another approach is the introduction of functional groups, such as ‐COOH, ‐OH, ‐NH_2,_ and ‐CN, rich in n → π* transitions, that could play a significant role in facilitating the afterglow process [29]. Last but not least, the so‐called clustering‐triggered emission (CTE), or clusteroluminescence, which occurs in such emitters despite the notable absence of π‐conjugated systems, appears to be a plausible additional reason for this non‐conventional emission [37, 38, 39]. All in all, non‐conjugated afterglow materials, which can be synthesized in large quantities and with ease of processing in optoelectronics, as well as in anti‐counterfeiting, remain challenging [40, 41, 42, 43, 44].

Herein, a series of straightforwardly up‐scalable, water‐processable, aliphatic terminal bisformamides (**BFA**) of general formula H(O═)CNH─(CH_2_)_n–_HNC(═O)H are set in as a new family of bright afterglow compounds and materials. As a representative example, the N,N'‐(hexane‐1,6‐diyl) bisformamide (*n* = 6, **BFAC6**) showed <τ> value a 0.40 s and a moderate φ of 13% in crystalline powder. While the presence of impurities has been ruled out by ^1^H‐^13^C NMR, x‐ray single‐crystal diffraction, and HPLC‐MS, we also noted the afterglow phenomenon in water‐processable polyvinyl alcohol (PVA) films (**BFAC6@PVA**) as well as after doping with N, N, N′, N′‐tetramethylbenzidine (TMB), lengthening more than 2‐fold <τ> and fairly keeping the other photoluminescence features.

Studies based on temperature‐dependent x‐ray analysis, time‐resolved electronic spectroscopy, and electrochemical impedance spectroscopy suggest that the unusual long‐lived emission could be attributed to the rigid packing and robust intermolecular interactions ruled by H‐bonding that result in an emitting excited state attributed to a H‐trapped emissive state in both **BFAC6** itself and **BFAC6@PVA** materials. This phenomenon is here potentially associated with CTE, or clusteroluminescence, since our system is electron rich and present a conformational rigidity—ruled by H‐bonding. Moreover, **BFAC6@PVA** materials can be applied for decoration, anti‐counterfeiting, and color down‐conversion in white‐emitting hybrid light‐emitting diodes (WHLEDs), showing promising prospects in these fields of application. What is more, the loss of the emission features could be attributed to the loss of crystallinity over time that can be restored upon gentle thermal treatment, enabling a straightforward recovery of the crystalline and emission features of these materials. Overall, this work headlines the **BFA** family as a promising route toward up‐scalable, water‐processable, stable, afterglow materials on simple non‐conjugated chemical materials.

## 2 Results and Discussion

### 2.1 Synthesis and Characterization

**FIGURE 1:**
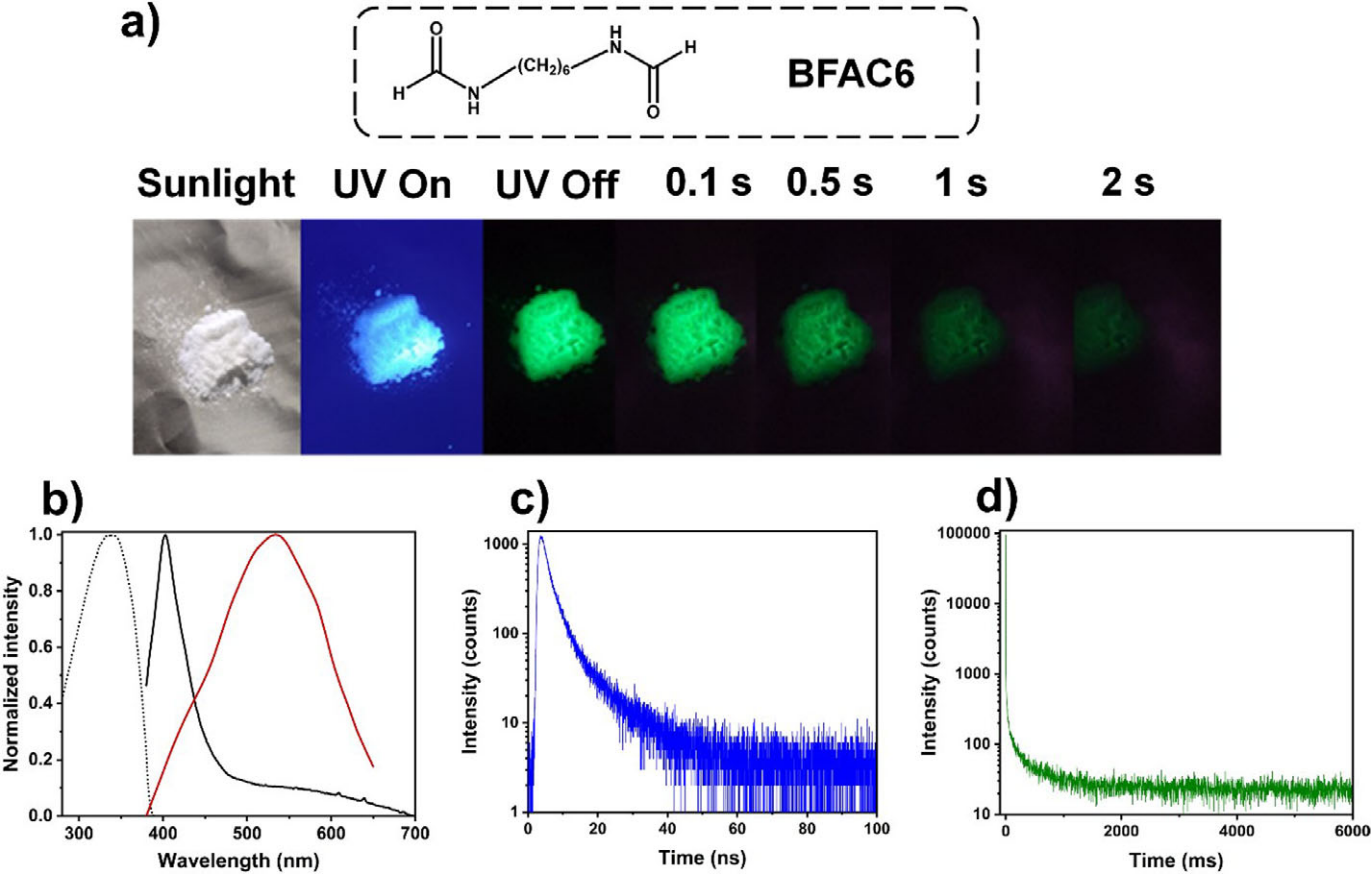
(a) Chemical structure of **BFAC6** and afterglow pictures of **BFAC6** crystals; (b) Excitation (dotted black line), prompt emission (full black line, λ_Exc_ = 370 nm), and delayed emission (full red line, λ_Exc_ = 370 nm) spectra of **BFAC6**; (c) Decay curve of the emission band at 400 nm of **BFAC6** excited at 375 nm; (d) Decay curve of the emission band at 550 nm of **BFAC6** excited at 370 nm.

This **BFA** family can be easily prepared by heating the respective aliphatic ethylene diamine in ethyl formate, reaching high reaction yields even in gram scale—see supporting information for details [45, 46]. Hereafter, we focus on the **BFAC6** derivative as a representative member of this family (Figure 1a), while others are discussed in the last section of this work for reference purposes. Specifically, the ^1^H and ^13^C NMR spectra of **BFAC6** at room‐ and high temperatures (Figures S1 and S2) [47] indicated the absence of impurities and the presence of two isomers *cis* and *trans*. The infrared spectrum of **BFAC6** (Figure S3) shows two neighboring bands at around 3273 and 3184 cm^−1^ due to the NH_2_ asymmetric and symmetric stretching vibrations, respectively. As well, a band at 2935 cm^−1^ due to CH stretching is also noted along with the bands related to the carbon‐oxygen double bond stretching at around 1715, 1697, and 1684 cm^−1^. More interestingly, **BFAC6** powder was found to exhibit a long afterglow for several seconds in the crystalline solid state at room temperature upon excitation with a 365 nm UV lamp (Figure 1a). This observation prompted us to carefully study its photophysical properties. In short, excitation at 370 nm results in two distinct emission bands. On the one hand, a relatively narrow emission band centered at around 400 nm with a full width at half maximum (FWHM) of 50 nm (Figure 1b) and a short‐lived emission (<τ> = 5.8 ns) (Figure 1c) was noted. On the other hand, the emission spectrum displays a very broad and featureless band centered (FWHM > 150 nm) at 550 nm (Figure 1b). This emission band is associated with a very long <τ> of 0.398 s at 370 nm excitation (Figure 1d) and, in turn, it is referred to an afterglow luminescence phenomenon [34, 48, 49, 50, 51, 52, 53, 54]. This overall emission exhibits a φ of 13%. Noteworthy, the emission spectra of **BFAC6** were recorded upon different excitation wavelengths, from 280 to 370 nm (Figure S4). In each case, the dual emission is observed, and at high excitation energy, the broad band at 550 nm is slightly higher in intensity.

To further understand the origin of this luminescence phenomenon, the influence of intermolecular interactions in BFAC6 was evaluated. To this end, the photophysical properties of BFAC6 in aqueous solutions were firstly investigated (Figure S5). The absorption spectrum of a dilute water solution (1 mm) of BFAC6 exhibits only one sharp absorption band due to the formamide group at 210 nm. However, as the concentration of BFAC6 increases, the absorption bathochromically shifts, showing a broadened absorption band centered at 240 nm at 0.1 m. At this concentration, additional absorption bands at 280 and 320 nm emerge, which may be considered to result from dense intermolecular interactions and molecular aggregation [48]. The luminescence of BFAC6 in aqueous solution is dramatically enhanced with concentration up to 0.8 m [25, 42]. In line with these results, the relationship between the luminescence properties of BFAC6 and its aggregation state was confirmed by single‐crystal XRD (Tables S1–S4).

**FIGURE 2:**
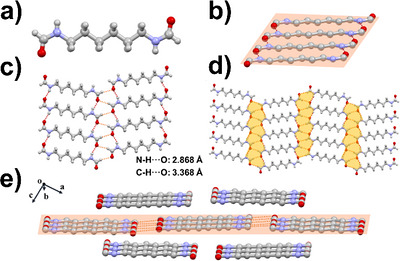
Crystal structure of **BFAC6**. (**a)** Representation of a single molecule of **BFAC6**; (b) Representation of the planar layer arrangement of **BFAC6** through intermolecular hydrogen bonding; (c) intermolecular hydrogen bonds in the **BFAC6** solid‐state structure; (d) Representation of four molecular strips highlighting the head‐on areas of the tight network of intermolecular hydrogen bonds; (e) Interlayer stacking in **BFAC6** crystals, hydrogens omitted for clarity (N─H···O: yellow dash, C─H···O: orange dash).

In detail, **BFAC6** crystallizes in the monoclinic P21/n space group, and the molecules are arranged in layers (Figure 2a,b). Within a layer, the molecules are laid out in neighboring strips with interdigitation between the strips. The molecules are connected through N─H···O hydrogen bonds (Figure S6) with a donor‐acceptor distance of 2.857 Å to the two adjacent molecules within the strips, while between two strips, short C─H···O contacts establish further connection with surrounding molecules to form the interdigitated array (Figure 2c) [55]. The strips of amides are tethered by N─H···O (perpendicular to the molecular chains) and C─H···O (along the chain), forming an interdigitated hydrogen‐bonding network through self‐assembly (Figure 2d), which might serve as a platform to form clusters, stabilize excitons, and therefore promote the above‐mentioned emission behavior. The adjacent hydrogen‐bonding channels show good coplanarity, disposed in anti‐parallel form into layers (Figures 2, S5). The molecules are tightly compacted in layers along the (0,1,3) crystal plane, with an interlayer distance of 3.35 Å (Figure 2e and Figure S6). These multiple intermolecular interactions provide a rigid environment restricting the molecular motions that might enable radiative deactivation. Despite the lack of *π–π* stacking, intermolecular interactions, such as N─H···O and C─H···O between the formamide groups, lead to a close arrangement which possibly serves as an extended H‐trapped emissive state [56, 57, 58]. Meanwhile, the rigid conformation and high crystallinity protect the material from undesirable humidity and molecular oxygen.

### 2.2 BFAC6 in Polymer Coatings

**FIGURE 3:**
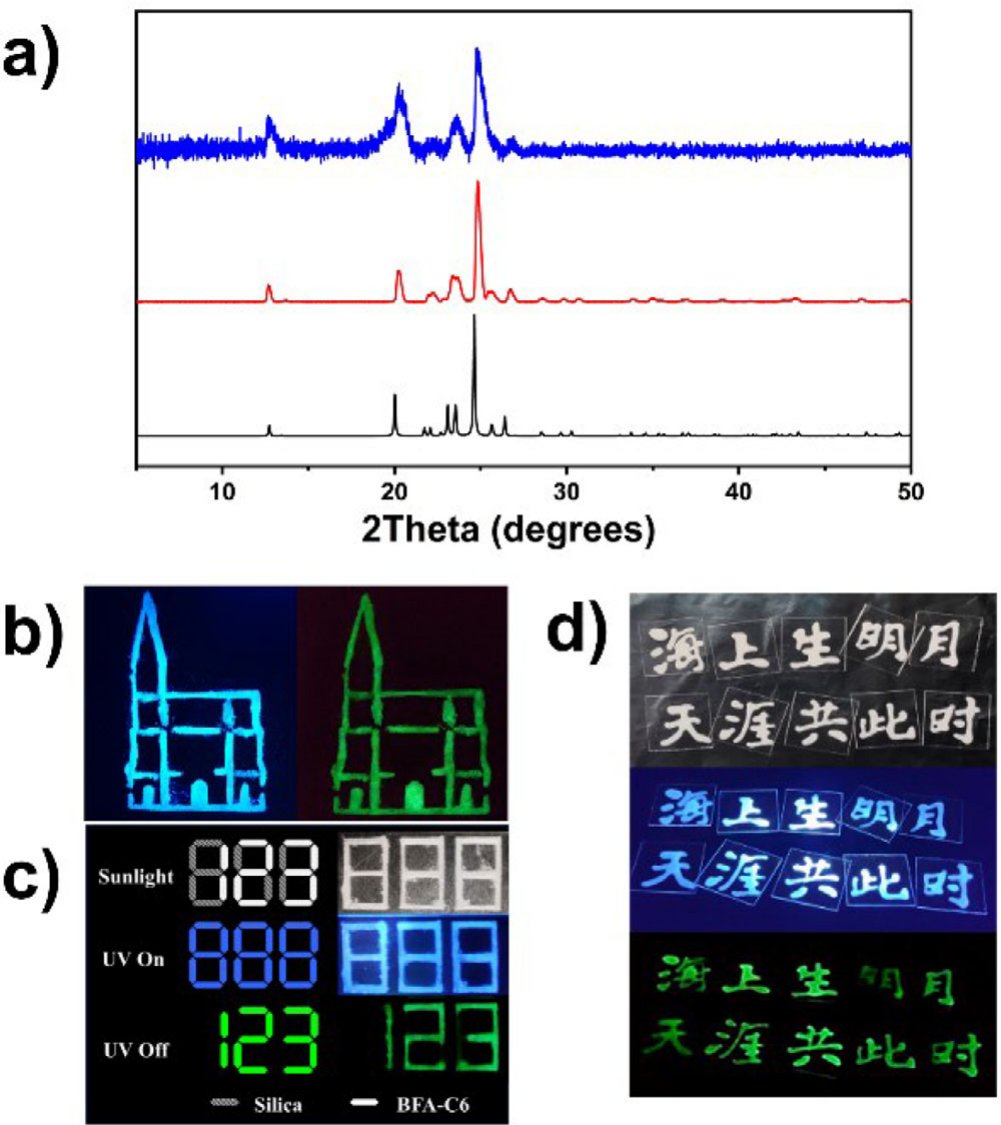
(a) PXRD patterns of **BAFC6@PVA** (blue line), **BFAC6** in powder state (red line), and simulated pattern (black line); (b) Drawing of the Strasbourg cathedral made by the powder of **BFAC6** (left: UV on, right: UV off); (c) Illustration of **BFAC6@PVA** ink used for anti‐counterfeiting purposes. (d) Poem from “Looking at the Moon and Thinking of One Far Away” by Chinese poet Zhang Jiuling, written with **BFAC6@PVA** film under sunlight (top), UV light (middle), and after turning off the UV light (bottom). English translation: “Over the sea has risen the bright moon, being far apart we share this moment”.

To integrate **BFAC6** based materials into applications, such as decoration, anti‐counterfeiting, and color down‐conversion in HLEDs, **BFAC6** was embedded into polyvinyl alcohol (PVA, Mw = 146,000–186,000 g/mol) in a mass weight ratio of 1:1.4 in a water‐based ink that form **BFAC6@PVA** films upon ambient drying—see supporting information for details. As shown in Figure 3a, the **BFAC6@PVA** films show a similar x‐ray pattern of the neat powder of **BFAC6**, suggesting the presence of large crystalline micro‐domains in the films, while the PVA polymer might be just acting as a supporting matrix. Indeed, the photoluminescence behavior is similar between powder and thin films (Figure S7). Here, the expected blue short‐living emission (400 nm, <τ> = 5.9 ns) and the green–yellow long‐living emission (550 nm, <τ> = 0.428 s) (Figure S7), together with the same φ of 13%, were clearly observed in both the films and the brushed drawings, as well as in anti‐counterfeiting applications. (Figure 3b–d).

**FIGURE 4:**
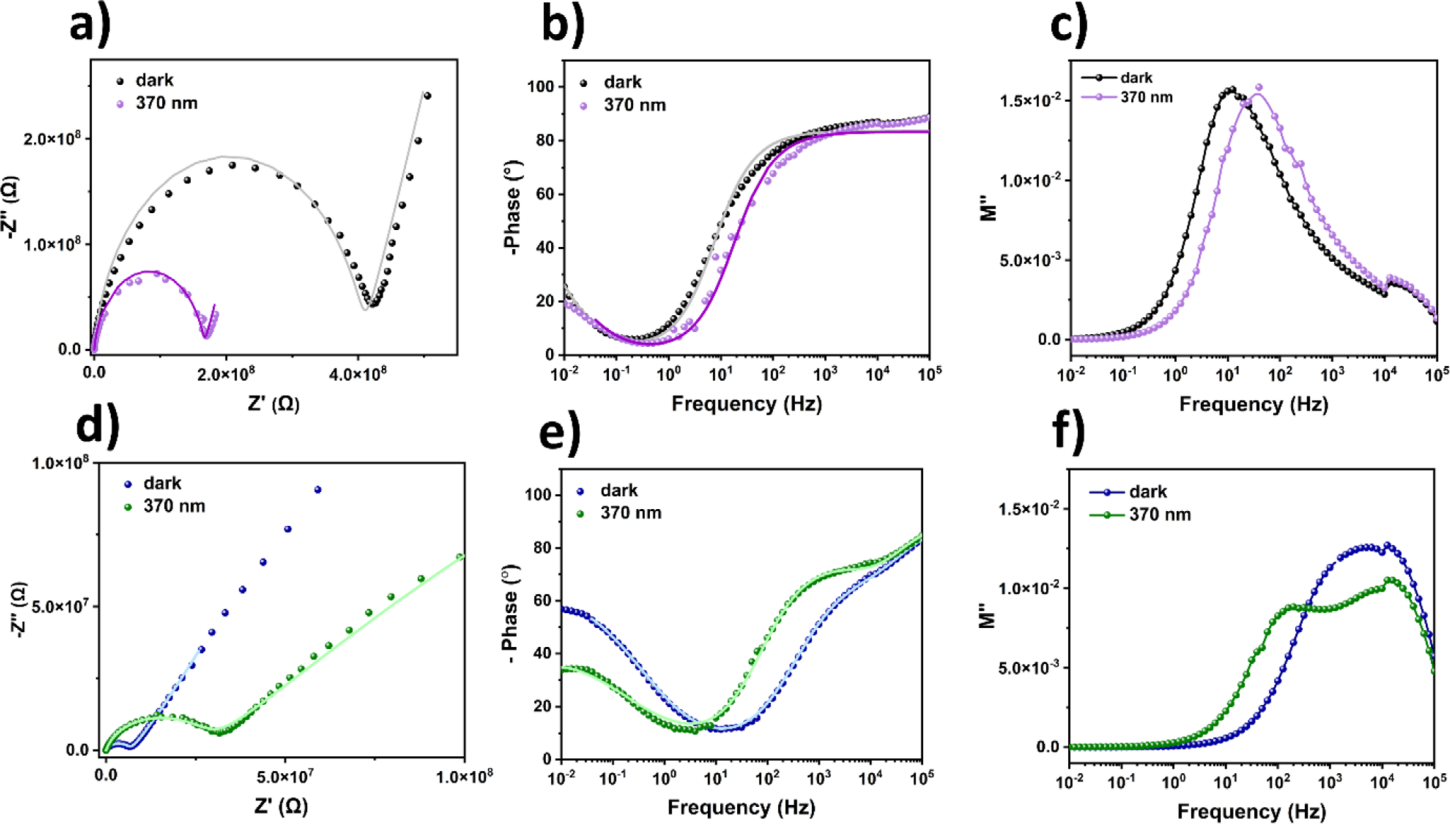
Nyquist (a,d), Bode plots (b,e), imaginary part of electric modulus (c,f) of PVA (top) and BFAC6@PVA (bottom) films under dark and UV‐illuminated (370 nm) conditions.

Furthermore, we capitalized on the PVA films to investigate the role of H‐transfer in the nature of the excited state using electrochemical impedance spectroscopy (EIS) measurements under dark and 370 nm UV illumination conditions (Figure 4). The equivalent electric circuit schemes used for fitting of Nyquist and phase angle plots are shown in Figure S8.

As discussed above, the long‐living emission in the **BFAC6** molecule could be tentatively attributed to a charge trapping event in stable, hydrogen‐bonded states that act as energy wells [59]. Under 370 nm excitation, one plausible scenario involves H‐coupled electron transfer strengthening the hydrogen bonding between **BFAC6** molecules within a crystalline domain [60]. At the same time, because **BFAC6** is a non‐aromatic molecule, CTE, also referred to as clusteroluminescence, should also be considered in line with the work of Yuan and coworkers and that of Tang and coworkers [34, 37, 38, 39, 54] In this picture, emission can emerge in the solid state or in concentrated systems through clustering of electron‐rich units, especially heteroatom‐containing motifs, together with conformational rigidification and restricted motion within the aggregate. Since these conditions are likely fulfilled in the **BFAC6@PVA** films, the present data are consistent with an open mechanistic picture in which hydrogen‐bonded clustering stabilizes long‐lived emissive or trapped states.

To test this hypothesis, we first studied reference neat PVA films that show a reduced resistance from 405 MΩ in the dark to about 166 MΩ under illumination. However, its dielectric response changes only modestly, with the main relaxation peak in the imaginary part of the electric modulus spectrum (M″), shifting from ∼12 to ∼39 Hz, as shown in Figure 4c. This change of behavior could be attributed to slightly enhanced mobility of protons or ions along the H‐bond network in PVA [61]. Having established our reference, the Nyquist plot of **BFAC6@PVA** films in the dark reveals a small semicircle followed by a pronounced Warburg‐type diffusion tail (Figure 4d). The equivalent circuit that best fits this state includes two branches: one at high frequency (*R*_1_ = 416 kΩ) and one dominant low‐frequency branch (*R*_2_ = 6.33 MΩ), together with a Warburg diffusion element, consistent with diffusion‐influenced and distributed charge transport [62, 63]. Upon UV illumination, an increase in resistance is observed: *R*_1_ increases to 599 kΩ, and *R*_2_ rises to 27.9 MΩ. This behavior differs sharply from that of neat PVA and points to stronger charge immobilization in the **BFAC6**‐containing film. Additionally, the Warburg element is replaced by a Gerischer impedance component, characterized by a rate constant *K*_a_ = 0.007 s^−^^1^, indicating that under illumination the low‐frequency response is better described by coupled diffusion‐reaction behavior than by pure diffusion alone [64]. Concurrently, a new low‐frequency feature emerges at 158 Hz in the M″ spectrum, indicating the emergence of an additional slow dielectric relaxation process. This observation, together with the rise in resistance, highlights efficient charge immobilization in trap states. These results indicate that UV exposure leads to photo‐stabilization of trapped charges, thereby suppressing rather than enhancing charge mobility. At the same time, the characteristic timescale derived from the Gerischer term, about 143 s, is much longer than the persistent luminescence lifetime, τ = 0.405 s. The impedance data therefore do not establish a direct one‐to‐one correspondence between the electrical relaxation process and the emissive state. Rather, they support a more cautious interpretation in which illumination generates longer‐lived trapped or stabilized charge states in a hydrogen‐bonded and possibly clustered environment. Within this framework, the emission may involve CTE, charge trapping, or a combination of both, but the present data do not allow an unambiguous distinction between these contributions. Finally, according to the work of Peng, Zhang and coworkers, the formation of structural water could have been considered as well [65, 66]. Nevertheless, this possibility has been ruled out because (i) this phenomenon typically occurs upon sample dilution, (ii) no structured water was observed in the SCXRD structures, and (iii) the crystalline aggregates are suggested to be protecting the material from undesirable humidity—*vide supra*.

As a next step, we investigated the application of **BFAC6** doped with 1 wt.% of N,N,N‘,N‘‐tetramethylbenzidine (**TMB‐BFAC6**) that can act as a H‐donor assisting molecule to increase the afterglow emission efficiency [67, 68].

At first, PXRD measurements ruled out the effect of different crystallization rates on the final crystalline properties of **TMB‐BFAC6** prepared via (i) uncontrolled cooling down from the melting point (m.p.) to room temperature (r.t.), (ii) slow and controlled cooling down from m.p. to r.t., using 10°C steps, (iii) fast cooling down from m.p. to −78°C and iv) ultra‐fast cooling down from m.p. to −196°C (Figure S9). The PXRD experiments lead to identical patterns for the four samples, and the Rietveld refinement revealed that **BFAC6** crystallizes according to the same monoclinic crystalline system and P21/n space group (Rietveld weighted profile, *R*_wp_ value = 4.1).

**FIGURE 5:**
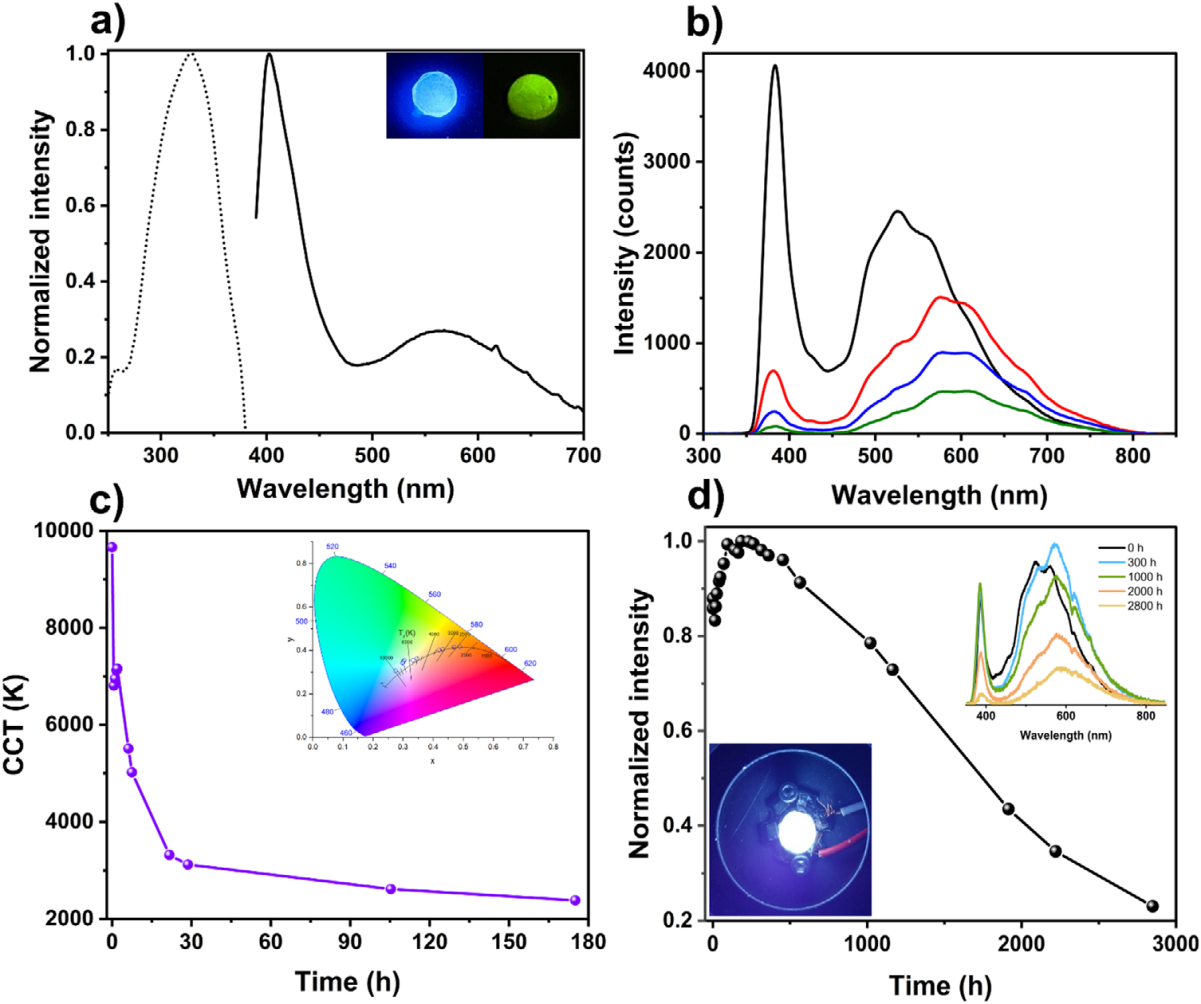
(a) Excitation (dotted line, λ_Em_ = 400 nm) and emission (full line, λ_Exc_ = 370 nm) spectra of **TMB‐BFAC6@PVA**. Inset: pictures of the coatings under 365 nm irradiation (left) and after turning off the excitation light source (right); (b) **TMB‐BFAC6@PVA** device emission spectra over time at 200 mA: t_0_ (black line), 22 h (red line), 105 h (blue line), 175 h (green line); (c) CCT of the **TMB‐BFAC6@PVA** device over time at 200 mA. Inset: *x, y* CIE coordinates plot of the device; (d) emission intensity decay profile of **TMB‐BFAC6@PVA** over time at 20 mA of applied current (Inset—bottom, left: picture of the WHLED; Inset—top, right: emission spectra of **TMB‐BFAC6@PVA** over time upon UV irradiation at 20 mA—see legend). All the data were acquired under ambient conditions.

The photophysical properties of **TMB‐BFAC6** in powder state were studied (Figure S10), and, in short, the φ of 13% associated to a prompt <τ> of 4.8 ns at 400 nm and a long <τ> of 0.910 s at 550 nm. This also points to an emission related to H‐trapped emitting excited states. Likewise, **TMB‐BFAC6@PVA** coatings kept the same PXRD pattern and the emission behavior with a short‐living high‐energy emission band (5.1 ns at 400 nm) and a long‐living lower‐energy emission (0.90 s at 550 nm) as shown in Figure 5a and Figure S11.

### 2.3 TMB‐BFAC6@PVA as Down‐Converting Materials in Lighting

The well‐defined long‐lived emission band of the **TMB‐BFAC6@PVA** coatings encouraged us to apply them as proof‐of‐concept color down‐converting filters in HLEDs that are placed on top of an unmodified 380 nm pumping LED chip—see supporting information for more details [69, 70, 71]. At first, the device stability was studied at a high applied current of 200 mA under ambient conditions. In line with the photoluminescence studies, Figure 5b shows that the emission of these devices consisted of an emission band at 390 nm and a broad emission band centered at *ca*. 540 nm. This results in a cold white light output, associated to moderate *x*, *y* CIE color coordinates of 0.28, 0.30, accompanied by a competitive color rendering index (CRI) of 85 and correlated color temperature (CCT) of 9000 K– Figure 5c. Over time, the variation in the relative contributions of the high‐energy band and the yellow band gradually leads to a warmer white light emission after 6 h (CCT = 5500 K), without affecting the CRI and showing (*x, y*) CIE color coordinates of 0.33, 0.34, that are very close to those of a perfect white light. However, the emission band intensity reaches half of its initial emission after 10 h (Figure S12), which is translated to a device lifetime of 10 h at 200 mA. In order to refine the device stability, such devices were run at different applied currents in a range of 10–200 mA—Figure S13. The expected emission response is similar regardless of the applied current, while the maximum luminous efficiency value of 1.7 lm/W is noted at 20 mA of applied current. Therefore, analogue stability tests upon continuous irradiation were run at 20 mA (Figure 5d), achieving a device lifetime of ca. 1,800 h. Like the device driven at a high applied current, a gradual ∼10 nm bathochromic shift of the low‐energy band leads to white color corruption over time.

**FIGURE 6:**
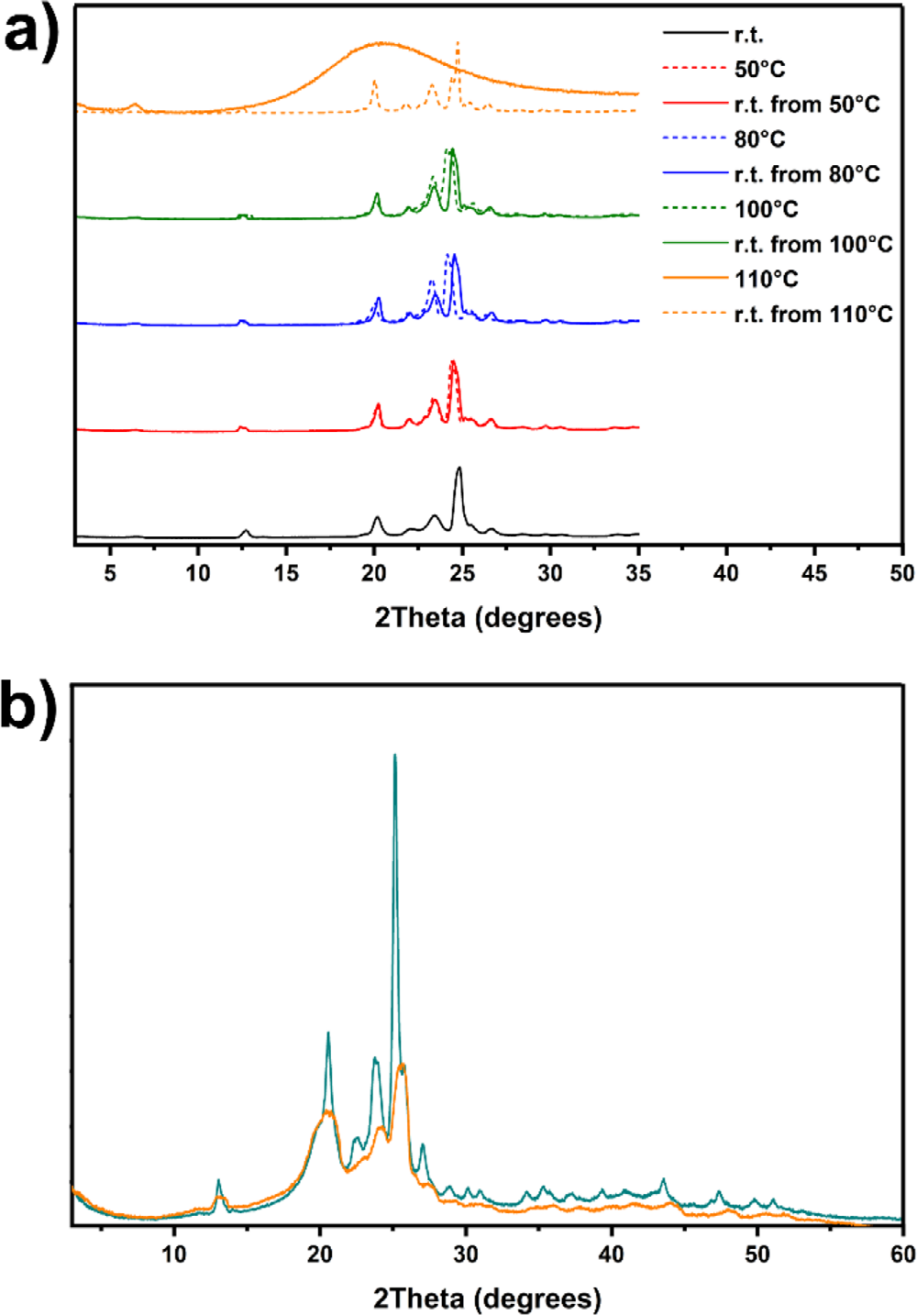
(a) PXRD pattern of TMB‐BFAC6@PVA in powder upon heating up at different temperatures and cooling down the powder sample to room temperature; (b) PXRD patterns of TMB‐BFAC6@PVA polymer coatings before and after continuous irradiation at λ_Exc_ = 380 nm.

To understand the emission changes over time, the φ changes of **TMB‐BFAC6@PVA** coatings were measured after use, showing a decrease down to 4% from the initial 13%. This suggests the formation of either degradative species and/or changes in the crystallinity features of the coatings. The latter was confirmed by comparing the XRD patterns of the coatings before and after irradiation (Figure 6). Here, the irradiation stress results in an overall broadening of the PXRD pattern, suggesting a transition toward an amorphous system in which we presume that the H‐transfer/CTE emission mechanism is hampered. Thus, the irradiated coatings were heated at temperatures of 50°C, 80°C, 100°C, and 110°C; and cooled back to room temperature between each heating process. In this thermal healing process, a total recovery of the emission features (φ = 13%) along with the crystallinity of the fresh **TMB‐BFAC6@PVA** coatings was noted. Thus, these results prompt us to state that bis‐formamide‐based emitters could lead to reusable materials using a quick and gentle temperature treatment; a characteristic that is barely met in the literature [72]. Interestingly, the device at 20 mA does not present a meaningful temperature increase (<30°C), excluding the hypothesis of a thermal quenching process as a degradation mechanism at such device driving conditions.

In order to gain more insight on the reusability of our material, cyclability studies were performed over multiple heating (110°C)—cooling (room temperature) cycles (Figure S14a,b). Recrystallization produced reflexes at the same positions, albeit with a texture effect. After five repetitions, there was no indication of an alteration of the recrystallized structure. Moreover, the emission spectrum of **TMB‐BFAC6@PVA** over four cycles points out a recovery of the material upon a quick, gentle heating process. Interestingly, at high temperature, the appearance of broad emission peaks from the third cycle (Figure S14) is comparable to what is observed from the device emission profile after 22 h (Figure 5b), and these peaks are not observed at room temperature.

Finally, temperature‐dependent emission spectra were recorded from ca. −196°C (77 K) to ca. 121°C (395 K) (Figure S14c,d). The emission wavelengths remain unchanged regardless of the temperature applied, even though the signal at 77 K is slightly more structured and less broad.

### 2.4 Versatility in Bis‐Formamide‐based Emitters (BFACn)

Finally, we also studied the structures and optical properties of compounds with different carbon chain lengths. On modifying the carbon chain from C2 to C12 (Figures S15–S20 and Tables S5‐S26), the luminescence of resulting the **BFACn** compounds showed a hypsochromic shift from 550 to 485 nm (Figure S20) with φ < 2% and <τ> ranging between 0.102 and 0.162 s, featuring a decreasing trend for chains shorter and longer than C6 with **BFAC6** at the center in line with the afterglow performance (Table S27). To rationalize the phenomenon, crystal structures of **BFACn** compounds were studied (Tables S5–S26). In particular, **BFAC2** and **BFAC12** were selected because of their short and long length carbon chain, and **BFAC7** was chosen to evaluate the difference between even and odd numbers in the carbon chain. **BFAC2** crystallizes in the orthorhombic Pbca space group and exhibits a non‐planar molecular geometry in the solid state and relatively loose molecular packing (Figure S18). The short aliphatic chain might cause less orderly molecular arrangement and thus affect the electron distributions in the crystalline structure and cause a stronger non‐radiative decay. In contrast, **BFAC12** crystalizes in triclinic system P‐1 space group, showing an intermolecular C─H···O interaction with only two adjacent molecules (Figure S19), indicating that the long carbon chain affects the arrangement of the hydrogen‐bonding network and leads to inferior photoexcitation efficiency.

Regarding **BFAC7**, it shows a long‐lived emission centered at around 500 nm (Figure S22) with <τ> of 0.162 s. Generally speaking, even‐numbered bisformamides show a better crystalline tendency than odd‐numbered ones, as it is also confirmed here (Tables S12–S14) [73]. In **BFAC6**, the two formyl groups at the end of the aliphatic chain present an antiparallel orientation, while their orientation is parallel for odd‐numbered compounds, as for instance in **BFAC7** (Figure S20). In addition, **BFAC6** has distinct centrosymmetric symmetry with a more rigid array, whereas **BFAC7** is C2 symmetric.

The **BFACn** compounds also show a non‐unexpected melting point alternation for odd and even chains, in line with the difference in spatial structure (Table S16) [72]. Among the **BFACn** compounds (*n* = 2, 6, 7, 12), **BFAC6** has the highest melting point, suggesting a higher symmetry of the crystal structure, which is in agreement with its stronger emission. H‐bonded crystals, such as **BFAC6**, are considered to have high melting points because H‐bonding holds the molecules together in the crystal. Finally, **BFAC6** also exhibits a lower entropy change on melting (Figure S23) compared to **BFAC7** [73]. This can be explained if one considers the strong intermolecular H‐bonds between **BFAC6** molecules [74].

## 3 Conclusion

This work demonstrates a readily accessible, straightforwardly up‐scalable and water‐processable aliphatic terminal bisformamide emitter family that features both short‐ and long‐lived luminescence properties, resulting in an afterglow response. Among them, **BFAC6** presents <τ> of 0.40 s and overall φ of 13%.

These features are related to its robust crystallinity and intermolecular interactions ruled by H‐bonding, in both powder and PVA films/coatings. Taken together, the results support a picture in which these structural factors stabilize long‐lived excited or trapped states. This interpretation is based on the combined photophysical data, EIS measurements under dark and illumination conditions in polymer coatings, as well as doping with 1% TMB, which further enhances the H‐transfer behavior of the material (*i.e*., a > 2‐fold longer excited‐state lifetime). In addition, in line with the state of the art in the field of afterglow luminescence, CTE is also suggested to contribute to this non‐conventional emission. The present data therefore support an open mechanistic picture in which hydrogen‐bond‐assisted charge trapping and CTE may both contribute to the observed afterglow. Next, the materials were applied in decoration, anti‐counterfeiting, and especially as down‐converting filters for WHLEDs implementation. What is more, the temperature‐driven healing process of the irradiated samples shows the recovery of the crystalline along with the emission features, making it an enthralling candidate for such applications, especially in terms of sustainability, scalability, and recovery.

Altogether, the present findings provide a better understanding of the emitting mechanism of such materials as well as guidelines for developing low‐cost, sustainable, and applicable non‐conjugated organic materials displaying long‐lived luminescence. An ongoing work in the realm of such investigations relies on the potential of transient absorption spectroscopy measurements to extend our knowledge, particularly about the emission mechanism of such afterglow, non‐conventional emitters.

## 4 Experimental Section

### 4.1 Materials and Methods

Nuclear magnetic resonance (NMR) data were collected on a Bruker AVANCE III 400 (400 MHz) Spectrometer and Bruker AVANCE III 500 (500 MHz).

High‐resolution mass spectrometry (HRMS) data were obtained on a Thermo Fisher Exactive Plus EMR Orbitrap mass spectrometer using the electrospray ionization technique.

Infrared (IR) experiments were performed using SHIMADZU IRAffinity‐1S Fourier transform infrared spectrophotometer in the region of 4000–700 cm^−1^.

Elemental analyses (C, H, and N) were performed on a Thermo Scientific FLASH 2000 CHNS/O Analyzer.

Melting points were measured on a digital melting point apparatus from Büchi, Model B‐540, after a calibration certification with standards.

Single‐crystal x‐ray diffraction data for crystals were collected on a Rigaku Oxford SuperNova x‐RAY diffractometer system equipped with a Mo sealed tube (λ = 0.71073 Å). The structure was solved and refined using the Bruker SHELXTL Software Package. All hydrogen atoms were located in calculated positions and refined anisotropically.

Powder x‐ray diffraction (PXRD) data used in this work were obtained from a Miniflex diffractometer (Rigaku, Tokyo, Japan) with a copper source (1.54Å) and a silicon strip detector (D/teX Ultra, Rigaku), a goniometer radius of 150 mm; both Soller slits at 2.5°; a divergence slit fixed at 0.625°; an anti‐scatter screen; and a kβ filter of 0.06 mm nickel foil. The powder was placed on a zero‐background silicon wafer sample holder (cut 911), and the measurement was performed at room temperature using a step size of 0.5°/min.

The cyclability of the crystal structure was traced by x‐ray diffraction (SmartLab, Rigaku, Tokyo, Japan) in Bragg‐Brentano geometry using a 9 kW rotating anode source and a heating table (DCS 500, Anton Paar, Graz, Austria). Samples were heated from 30°C to 110 *°*C. Their diffractograms were recorded at both temperatures and in between, from 5* < *2*θ*/*°* < 60 at a resolution of 0.05° and a scan speed of 50 °/min.

Photoluminescence pictures were taken under a UV lamp using UV radiation of 365 nm. Excitation/emission spectra and time‐resolved measurements were recorded with a HORIBA FluoroLog‐3 and an Edinburgh FS5 spectrometer. A pulsed xenon lamp was used to excite the samples for the steady‐state measurements, and a photodiode was used for the time‐resolved measurements. The photoluminescence quantum yields were measured with an absolute photoluminescence quantum yield measurement integrating sphere (Hamamatsu, C11347‐11).

The impedance measurements were carried out using a potentiostat PGSTAT204, Metrohm Autolab with FRA32M module for impedance analysis. The PVA and **BFAC6** films were placed between two FTO electrodes. A frequency range of 0.01 Hz–150 kHz was used with an applied ac bias of 100 mV (no dc bias was applied). The system was illuminated using 370 nm LED irradiation of 50 mW cm^−2^ (WINGER Power LED Star) as pumping sources. The fit and simulation were done using Nova 2.1.5 software.

The HLEDs were fabricated by placing the down‐converting coating on top of an LED chip (380 nm, WINGER Power LED Star) with irradiation ranging between 10 and 200 mA, with 200 mA corresponding to a photon flux of 80 mW cm^−2^.

### 4.2 Synthesis and Preparation of BFACn Compounds and Corresponding Materials

**BFACn compounds** were prepared from diamino hexane and ethyl formamide following a reported procedure [45, 46, 73].

**BFAC6**: Yield: 91%. Melting point: 107.4‐108.5°C. ^1^H NMR (500 MHz, DMSO‐*d6*, 25°C, TMS): δ = 7.98(s, CHO), 7.91(s, CHO), 3.07(m, 4H, CH2), 1.39(m, 4H, CH2), 1.25(m, 4H, CH2). ^13^C ‐NMR (126 MHz, D_2_O, 25°C, TMS): δ = 164.93, 161.34, 41.21, 37.44, 31.29, 29.40, 26.45, 26.39, 25.97. HRMS (ESI^+^, MeOH, *m/z*): calcd for [M + H]^+^, 173.127; found, 173.128. Anal. Calcd for (C8H16N2O2): C, 55.79; H, 9.36; N, 16.27. Found: C, 55.79; H, 9.38; N, 16.22.

### 4.3 Characterization of BFACn Compounds

**BFAC2**: Yield: 88%. Melting point: 107.0‐108.2°C (ref. 105°C). ^1^H NMR (500 MHz, DMSO‐*d6*, 25°C, TMS): δ = 3.15(m, 4H, methylene CH2), 7.67(s, NH), 7.86(s, CHO), 8.01(s, CHO), 8.04(s, NH). ^13^C ‐NMR (126 MHz, DMSO‐*d6*, 25°C, TMS): δ = 37.32, 161.85, 165.09. HRMS (ESI^+^, MeOH, *m/z*): calcd for [M + H]^+^, 117.066; found, 117.066. Anal. Calcd for (C4H8N2O2): C, 41.37; H, 6.94; N, 24.13. Found: C, 41.36; H, 6.95; N, 23.97.

**BFAC4**: Yield: 86%. Melting point: 92.8‐94.1°C (ref. 92.5°C). ^1^H NMR (500 MHz, DMSO‐*d6*, 25°C, TMS): δ = 7.99(s, CHO), 7.91(s, CHO), 3.07(m, 4H, CH2), 1.40(m, 4H, CH2). ^13^C ‐NMR (126 MHz, DMSO‐*d6*, 25°C, TMS): δ = 164.97, 161.41, 40.93, 37.18, 37.10, 26.93. HRMS (ESI^+^, MeOH, *m/z*): calcd for [M + H]^+^, 145.097; found, 145.097. Anal. Calcd for (C6H12N2O2): C, 49.99; H, 8.39; N, 19.43. Found: C, 49.97; H, 8.36; N, 19.35.

**BFAC5**: Yield: 75%. Melting point: 61.3‐62.7°C (ref. 64.5°C). ^1^H NMR (500 MHz, DMSO‐*d6*, 25°C, TMS): δ = 7.99(s, CHO), 7.91(s, CHO), 3.05(m, 4H, CH2), 1.40(m, 4H, CH2), 1.25(m, 2H, CH2). ^13^C ‐NMR (126 MHz, DMSO‐*d6*, 25°C, TMS): δ = 164.93, 161.37, 41.19, 37.42, 30.96, 29.08, 24.14, 23.68. HRMS (ESI^+^, MeOH, *m/z*): calcd for [M + H]^+^, 159.112; found, 159.112. Anal. Calcd for (C7H14N2O2): C, 53.15; H, 8.92; N, 17.71. Found: C, 52.13; H, 8.79; N, 17.35.

**BFAC7**: Yield: 90%. Melting point: 73.6‐75.8°C (ref. 77.5°C). ^1^H NMR (500 MHz, DMSO‐*d6*, 25°C, TMS): δ = 7.98(s, CHO), 7.91(s, CHO), 3.05(m, 4H, CH2), 1.39(m, 4H, CH2), 1.25(m, 6H, CH2). ^13^C ‐NMR (126 MHz, DMSO‐*d6*, 25°C, TMS): δ = 164.92, 161.33, 41.26, 37.48, 31.29, 29.41, 28.79, 28.72, 26.76, 26.27. HRMS (ESI^+^, MeOH, *m/z*): calcd for [M + H]^+^, 187.144; found, 187.144. Anal. Calcd for (C9H18N2O2): C, 58.04; H, 9.74; N, 15.04. Found: C, 57.85; H, 9.73; N, 14.90.

**BFAC8**: Yield: 85%. Melting point: 91.6‐92.8°C (ref. 87°C). ^1^H NMR (500 MHz, DMSO‐*d6*, 25°C, TMS): δ = 7.98(s, CHO), 7.91(s, CHO), 3.06(m, 4H, CH2), 1.39(m, 4H, CH2), 1.25(m, 8H, CH2). ^13^C ‐NMR (126 MHz, DMSO‐*d6*, 25°C, TMS): δ = 164.91, 161.32, 41.28, 37.49, 31.34, 29.44, 29.09, 29.04, 26.75, 26.28. HRMS (ESI^+^, MeOH, *m/z*): calcd for [M + H]^+^, 201.158; found, 201.159. Anal. Calcd for (C10H20N2O2): C, 59.97; H, 10.07; N, 13.99. Found: C, 59.98; H, 10.14; N, 13.99.

**BFAC10**: Yield: 78%. Melting point: 97.9‐99.1°C. ^1^H NMR (500 MHz, DMSO‐*d6*, 25°C, TMS): δ = 7.98(s, CHO), 7.91(s, CHO), 3.06(m, 4H, CH2), 1.40(m, 4H, CH2), 1.25(m, 12H, CH2). ^13^C ‐NMR (126 MHz, DMSO‐*d6*, 25°C, TMS): δ = 164.89, 161.30, 41.28, 37.49, 31.37, 29.47, 29.41, 29.39, 29.15, 29.09, 26.81, 26.33. HRMS (ESI^+^, MeOH, *m/z*): calcd for [M + H]^+^, 229.191; found, 229.190. Anal. Calcd for (C12H24N2O2): C, 63.12; H, 10.59; N, 12.27. Found: C, 63.14; H, 10.67; N, 12.30.

**BFAC12**: Yield: 65%. Melting point: 102.7‐104.9°C. ^1^H NMR (500 MHz, CDCl3, 25°C, TMS): δ = 8.10(s, CHO), 7.96(s, CHO), 3.23(m, 4H, CH2), 1.45(m, 4H, CH2), 1.19(m, 16H, CH2). ^13^C ‐NMR (126 MHz, CDCl3, 25°C, TMS): δ = 164.62, 161.20, 41.76, 38.20, 31.21, 29.52, 29.50, 29.43, 29.41, 29.39, 29.17, 29.13, 29.08, 26.80, 26.77, 26.33. HRMS (ESI^+^, MeOH, *m/z*): calcd for [M + H]^+^, 257.222; found, 257.221. Anal. Calcd for (C14H28N2O2): C, 65.59; H, 11.01; N, 10.93. Found: C, 65.60; H, 11.02; N, 10.83.

### 4.4 Preparation of BFAC6@PVA Films for Decoration and Anti‐Counterfeiting Applications

**BFAC6** is dispersed in 5% PVA (polyvinyl alcohol, 5 g in 100 mL deionized water (50 mg/mL)) aqueous solution with a certain content (70 mg/mL, 0.4 m), and then a homogeneous solution was obtained after ultrasonication for 20 min. The solution was injected into a pen cartridge to yield security ink for painting.

The obtained **BFAC6@PVA** homogeneous solution is painted on a glass surface, dried naturally to form the film, then dried under 60°C in a vacuum oven overnight.

### 4.5 Preparation of TMB‐BFAC6

**TMB‐BFAC6** was obtained through a typical melt‐casting method in which the mixture of two compounds (10 mg of TMB for 1 g of BFAC6) were heated over the melting point of BFAC6 (150°C) under a nitrogen atmosphere and then cooled slow to room temperature at different rates: (i) slow cooling of 10°C steps to r.t., regular cooling to r.t. by stopping the heating, (iii) sudden cooling to −78°C, (iv) sudden cooling to −196°C.

### 4.6 Preparation of the Photon Down‐Converting Coatings, TMB‐BFAC6@PVA

A 80 mg·mL^−1^ polyvinyl alcohol (PVA, average Mw = 146,000–186,000 g/mol) solution in water was made by mixing 500 mg PVA in 10 mL of water. Prior to heating at 80°C and stirring overnight. 70 mg of the emissive material, **TMB‐BFAC6** was put in a vial, and 900 µL of the PVA solution in water was poured in the vial. The resulting suspension was heated at 80°C and stirred at 300 rpm for 30 min, until the powder is fully dissolved. The final suspension was allowed to cool down to r. t., and 300 µL of it were put in a well‐shaped Teflon mold. The suspension was left to dry for 48 h at ambient conditions, and the resulting dry, dome‐shaped coating was recovered. The thickness of the coating was measured using a Helios‐Preisser electronic outside micrometer (thickness measured: 1.49 ± 0.05 mm).

CCDC No. of BFAC2: CCDC 2479052 (BFAC2), 2479053 (BFAC4), 2150359 (BFAC6), 2099953 (BFAC7), 2150358 (BFAC8), 2099962 (BFAC10), 2099963 (BFAC12), contains the supplementary crystallographic data for this paper. These data can be obtained free of charge from The Cambridge Crystallographic Data Centre via www.ccdc.cam.ac.uk/data_request/cif and Fachinformationszentrum Karlsruhe Access Structures service.

## Funding

C.Z. and J.‐M.L. thank the ERC (Advanced Research Grant SUPRADAPT 290585), the ANR DYNAFUN grant No. ANR‐15‐CE29‐0009‐01, the University of Strasbourg Institute for Advanced Study (USIAS) and the CNRS (UMR 7006) for financial support. C.Z. acknowledges a doctoral fellowship from the China Scholarship Council. R.D.C. and S.F. acknowledge the European Union's Horizon 2020 research and innovation FET‐OPEN under grant agreement ARTIBLED No 863170. A.Z. thanks the Fonds der Chemischen Industrie for a Liebig Fellowship and TU Munich for support through the TUM Junior Fellow Funds. Y.A. and R.D.C. acknowledge the European Union's Horizon 2020 research and innovation CuMOF‐LED No. 896800.

## Conflicts of Interest

The authors declare no conflicts of interest.

## Supporting information




**Supporting File**: smll74099‐sup‐0001‐SuppMat.pdf.

## Data Availability

The data that support the findings of this study are available in the Supporting Information of this article.

## References

[1] S. Y. Chang , G. T. Lin , Y. C. Cheng , et al., “Construction of Highly Efficient Carbazol‐9‐yl‐Substituted Benzimidazole Bipolar Hosts for Blue Phosphorescent Light‐Emitting Diodes: Isomer and Device Performance Relationships,” ACS Applied Materials & Interfaces 10 (2018): 42723–42732, 10.1021/acsami.8b15084. 30360065

[2] T. Ito , H. Sasabe , Y. Nagai , Y. Watanabe , N. Onuma , and J. Kido , “A Series of Dibenzofuran‐Based n‐Type Exciplex Host Partners Realizing High‐Efficiency and Stable Deep‐Red Phosphorescent OLEDs,” Chemistry—A European Journal 25 (2019): 7308–7314, 10.1002/chem.201805907. 30741443

[3] S. M. A. Fateminia , Z. Mao , S. Xu , Z. Yang , Z. Chi , and B. Liu , “Organic Nanocrystals with Bright Red Persistent Room‐Temperature Phosphorescence for Biological Applications,” Angewandte Chemie International Edition 129 (2017): 12328–12332. 10.1002/anie.201705945 28771963

[4] A. Fermi , G. Bergamini , M. Roy , M. Gingras , and P. Ceroni , “Turn‐on Phosphorescence by Metal Coordination to a Multivalent Terpyridine Ligand: A New Paradigm for Luminescent Sensors,” Journal of the American Chemical Society 136 (2014): 6395–6400, 10.1021/ja501458s. 24725096

[5] X. Yang and D. Yan , “Long‐Afterglow Metal–Organic Frameworks: Reversible Guest‐Induced Phosphorescence Tunability,” Chemical Science 7 (2016): 4519–4526, 10.1039/C6SC00563B. 30155098 PMC6016333

[6] Z. Wang , C. Y. Zhu , S. Y. Yin , et al., “A Metal–Organic Supramolecular Box as a Universal Reservoir of UV, WL, and NIR Light for Long‐Persistent Luminescence,” Angewandte Chemie International Edition 58 (2019): 3481–3485. 30615238 10.1002/anie.201812708

[7] Z. Chai , C. Wang , J. Wang , et al., “Abnormal Room Temperature Phosphorescence of Purely Organic Boron‐Containing Compounds: The Relationship Between The Emissive Behavior and The Molecular Packing, and The Potential Related Applications,” Chemical Science 8 (2017): 8336–8344, 10.1039/C7SC04098A. 29619180 PMC5858747

[8] D. Sasikumar , A. T. John , J. Sunny , and M. Hariharan , “Access to the Triplet Excited States of Organic Chromophores,” Chemical Society Reviews 49 (2020): 6122–6140, 10.1039/D0CS00484G. 32794539

[9] Z. Yang , C. Xu , W. Li , et al., “Boosting the Quantum Efficiency of Ultralong Organic Phosphorescence up to 52 % via Intramolecular Halogen Bonding,” Angewandte Chemie International Edition 59 (2020): 17451–17455. 32638499 10.1002/anie.202007343

[10] E. Hamzehpoor and D. F. Perepichka , “Crystal Engineering of Room Temperature Phosphorescence in Organic Solids,” Angewandte Chemie International Edition 132 (2020): 10063–10067. 10.1002/anie.201913393 31725174

[11] R. Gavale , S. Kumar Pandit , and R. Misra , “Room Temperature Phosphorescence Driven by Mechanochromism,” Chemistry—An Asian Journal 20 (2025): 202401893, 10.1002/asia.202401893. 39985187

[12] B. Zhou , Q. Zhao , L. Tang , and D. Yan , “Tunable Room Temperature Phosphorescence and Energy Transfer in Ratiometric Co‐Crystals,” Chemical Communications 56 (2020): 7698–7701, 10.1039/D0CC02730H. 32579630

[13] S. Xu , W. Wang , H. Li , et al., “Design of Highly Efficient Deep‐Blue Organic Afterglow Through Guest Sensitization and Matrices Rigidification,” Nature Communications 11 (2020): 4802, 10.1038/s41467-020-18572-9. PMC7511363 32968080

[14] X. G. Yang , X. M. Lu , Z. M. Zhai , et al., “Facile Synthesis of a Micro‐Scale MOF Host–Guest with Long‐Lasting Phosphorescence and Enhanced Optoelectronic Performance,” Chemical Communications 55 (2019): 11099. 31460527 10.1039/c9cc05708k

[15] S. Cai , H. Shi , D. Tian , et al., “Enhancing Ultralong Organic Phosphorescence by Effective π‐Type Halogen Bonding,” Advanced Functional Materials 28 (2018): 1705045, 10.1002/adfm.201705045.

[16] Y. Mu , F.‐Y. Cao , X.‐Y. Fang , et al., “Tunable Full‐Color Room Temperature Phosphorescence of Two Single‐Component Zinc(II)‐Based Coordination Polymers,” Advanced Optical Materials 11 (2022): 2202402, 10.1002/adom.202202402.

[17] L. Favereau , C. Quinton , C. Poriel , T. Roisnel , D. Jacquemin , and J. Crassous , “Persistent Organic Room‐Temperature Phosphorescence in Cyclohexane‐*trans*‐1,2‐Bisphthalimide Derivatives: The Dramatic Impact of Heterochiral vs Homochiral interactions,” The Journal of Physical Chemistry Letters 11 (2020): 6426–6434. 32680427 10.1021/acs.jpclett.0c01816

[18] Y. Gong , G. Chen , Q. Peng , et al., “Achieving Persistent Room Temperature Phosphorescence and Remarkable Mechanochromism From Pure Organic Luminogens,” Advanced Materials 27 (2015): 6195–6201, 10.1002/adma.201502442. 26456393

[19] K. Patir and S. K. Gogoi , “Long Afterglow Room‐Temperature Phosphorescence From Nanopebbles: A Urea Pyrolysis Product,” Chemistry—An Asian Journal 14 (2019): 2573–2578, 10.1002/asia.201900454. 31044533

[20] W. Zhao , Z. He , and B. Z. Tang , “Room‐Temperature Phosphorescence from Organic Aggregates,” Nature Reviews Materials 5 (2020): 869–885, 10.1038/s41578-020-0223-z.

[21] Q. Y. Yang and J. M. Lehn , “Bright White‐Light Emission From a Single Organic Compound in the Solid State,” Angewandte Chemie International Edition 53 (2014): 4572–4577. 24677585 10.1002/anie.201400155

[22] M. Stanitska , D. Volyniuk , B. Minaev , H. Agren , and J. V. Grazulevicius , “Molecular Design, Synthesis, Properties, and Applications of Organic Triplet Emitters Exhibiting Blue, Green, Red and White Room‐Temperature Phosphorescence,” Journal of Materials Chemistry C 12 (2024): 2662–2698, 10.1039/D3TC04514E.

[23] Y. Zheng , Z. Li , and H. Zhang , “Visible‐Excitable Long‐Afterglow Material with Dual‐Mode Emission of Delayed Fluorescence and Room Temperature Phosphorescence,” Journal of Materials Chemistry C 12 (2024): 11506–11512, 10.1039/D4TC02024C.

[24] S. Wang , D. Wu , S. Yang , Z. Lin , and Q. Ling , “platform for colorful afterglow,” Materials Chemistry Frontiers 4 (2020): 1198–1205, 10.1039/D0QM00018C.

[25] Q. Li , Y. Tang , W. Hu , and Z. Li , “Fluorescence of Nonaromatic Organic Systems and Room Temperature Phosphorescence of Organic Luminogens: The Intrinsic Principle and Recent Progress,” Small 14 (2018): 1801560, 10.1002/smll.201801560. 30073754

[26] Y. Gao , H. Zhang , Y. Jiao , et al., “Strategy for Activating Room‐Temperature Phosphorescence of Carbon Dots in Aqueous Environments,” Chemistry of Materials 31 (2019): 7979–7986, 10.1021/acs.chemmater.9b02176.

[27] Y. Wang , S. Tang , Y. Wen , S. Zheng , B. Yang , and W. Z. Yuan , “Nonconventional Luminophores with Unprecedented Efficiencies and Color‐Tunable Afterglows,” Materials Horizons 7 (2020): 2105–2112, 10.1039/D0MH00688B.

[28] S. Zheng , T. Zhu , Y. Wang , T. Yang , and W. Z. Yuan , “Accessing Tunable Afterglows From Highly Twisted Nonaromatic Organic AIEgens via Effective Through‐Space Conjugation,” Angewandte Chemie 132 (2020): 10104–10108, 10.1002/ange.202000655. 32065715

[29] Q. Peng , H. Ma , and Z. Shuai , “Theory of Long‐Lived Room‐Temperature Phosphorescence in Organic Aggregates,” Accounts of Chemical Research 54 (2021): 940–949, 10.1021/acs.accounts.0c00556. 33347277

[30] X. Ma , C. Xu , J. Wang , and H. Tian , “Amorphous Pure Organic Polymers for Heavy‐Atom‐Free Efficient Room‐Temperature Phosphorescence Emission,” Angewandte Chemie 130 (2018): 11020–11024, 10.1002/ange.201803947. 29719096

[31] C. Li , M. Wu , Z. Z. Jacky , W. Y. B. Xu , and B. Z. Tang , “Suppressing Molecular Motions: A Pathway to Enhanced Organic Room‐Temperature Phosphorescence,” Chem 11 (2025): 102654.

[32] Z. An , C. Zheng , Y. Tao , et al., “Stabilizing Triplet Excited States for Ultralong Organic Phosphorescence,” Nature Materials 14 (2015): 685–690, 10.1038/nmat4259. 25849370

[33] S. Cai , H. Shi , J. Li , et al., “Visible‐Light‐Excited Ultralong Organic Phosphorescence by Manipulating Intermolecular Interactions,” Advanced Materials 29 (2017): 1701244, 10.1002/adma.201701244. 28714219

[34] Z. He , W. Zhao , J. W. Y. Lam , et al., “White Light Emission from A Single Organic Molecule with Dual Phosphorescence at Room Temperature,” Nature Communications 8 (2017): 416, 10.1038/s41467-017-00362-5. PMC5583377 28871160

[35] T. Zhang , Y. Jing , Z. Wang , et al., “Room‐Temperature Phosphorescence Based on Doping Systems: Material design, mechanisms, and applications,” Materials Chemistry Frontiers 10 (2026): 538–564, 10.1039/D5QM00552C.

[36] W. Ye , C. Huang , A. Lv , et al., “Rigid Ionic‐Bonding Networks Boosting Organic Room Temperature Phosphorescence,” Nature Communications 17 (2026): 1759, 10.1038/s41467-026-68468-3. PMC12913717 41582163

[37] J. Zhang , Z. Xiong , H. Zhang , and B. Z. Tang , “Emergent Clusteroluminescence from Nonemissive Molecules,” Nature Communications 16 (2025): 3910, 10.1038/s41467-025-59212-4. PMC12032425 40280920

[38] Z. Zhao , A. Li , and W. Z. Yuan , “Nonconventional Luminophores: Emission Mechanism, Regulation, and Applications,” Accounts of Chemical Research 58 (2025): 612–624, 10.1021/acs.accounts.4c00794. 39918223

[39] Q. Zhang , Z. Zhao , G. Yang , et al., “Synergistic Photoluminescence Enhancement in Nonaromatic Amino Acids and Sugars via Glycosylation,” Advanced Functional Materials 35 (2025): 2423603, 10.1002/adfm.202423603.

[40] S. Ferrara , S. H. Mejias , M. Liutkus , et al., “Designing Artificial Fluorescent Proteins: Squaraine‐LmrR Biophosphors for High Performance Deep‐Red Biohybrid Light‐Emitting Diodes,” Advanced Functional Materials 32 (2022): 2111381, 10.1002/adfm.202111381.

[41] D. Gutiérrez‐Armayor , Y. Atoini , D. Van Opdenbosch , C. Zollfrank , M. Nieddu , and R. D. Costa , “Simple Sol‐Gel Protein Stabilization Toward Rainbow and White Lighting Devices,” Advanced Materials 36 (2024): 2311031, 10.1002/adma.202311031. 38597244

[42] J. He , S. Yang , K. Zheng , Y. Zhang , J. Song , and J. Qu , “One‐Pot Synthesis of Dispersible Thermally Stable Organic Downconversion Materials under DBU Catalyzation for High Performance Hybrid‐LED Lamps,” Green Chemistry 20 (2018): 3557–3565, 10.1039/C8GC01390J.

[43] A. Luridiana , G. Pretta , D. Chiriu , et al., “A Facile Strategy for New Organic White LED Hybrid Devices: Design, Features and Engineering,” RSC Advances 6 (2016): 22111–22120, 10.1039/C6RA00999A.

[44] M. Mosca , R. Macaluso , and I. Crupi , “Hybrid Inorganic‐Organic White Light Emitting Diodes,” in Polymers for Light‐Emitting Devices and Displays (Wiley‐Scrivener Publishing, 2020), 197, 10.1002/9781119654643.

[45] A. D. Fernando Pulle , H. Frisch , and B. T. Tuten , “Direct Chromophore Integration into Polymer Backbones via Rhodanine Step‐Growth Chemistry,” Polymer Chemistry 16 (2025): 4215–4221, 10.1039/D5PY00297D.

[46] A. C. Boukis and M. A. R. Meier , “Data Storage in Sequence‐Defined Macromolecules via Multicomponent Reactions,” European Polymer Journal 104 (2018): 32–38, 10.1016/j.eurpolymj.2018.04.038.

[47] Q. Chen , X. Liu , K. Xu , C. Song , W. Zhang , and P. Wang , “Phase Behavior and Self‐Assembly of poly[ N ‐vinylformamide‐ co ‐(acrylic acid)] Copolymers under Highly Acidic Conditions,” Journal of Applied Polymer Science 109 (2008): 2802–2807, 10.1002/app.28271.

[48] Z. Yin , M. Gu , H. Ma , et al., “Molecular Engineering Through Control of Structural Deformation for Highly Efficient Ultralong Organic Phosphorescence,” Angewandte Chemie International Edition 133 (2021): 2086–2091. 10.1002/anie.202011830 32902079

[49] K. Nagarajan , A. R. Mallia , V. S. Reddy , and M. Hariharan , “Access to Triplet Excited State in Core‐Twisted Perylenediimide,” The Journal of Physical Chemistry C 120 (2016): 8443–8450.

[50] H. Shi , L. Bian , M. Gu , et al., “Colour‐Tunable Ultra‐Long Organic Phosphorescence of A Single‐Component Molecular Crystal,” Nature Photonics 13 (2019): 406–411.

[51] C. Zhou , S. Zhang , Y. Gao , et al., “Ternary Emission of Fluorescence and Dual Phosphorescence at Room Temperature: A Single‐Molecule White Light Emitter Based on Pure Organic Aza‐Aromatic Material,” Advanced Functional Materials 28 (2018): 1802407, 10.1002/adfm.201802407.

[52] X. Yang , G. I. N. Waterhouse , S. Lu , and J. Yu , “Recent Advances in the Design of Afterglow Materials: Mechanismss, Structural Regulation Strategies and Applications,” Chemical Society Reviews 52 (2023): 8005–8058, 10.1039/D2CS00993E. 37880991

[53] L. Zang , W. Shao , O. Bolton , et al., “Polarity‐Induced Dual Room‐Temperature Phosphorescence Involving the T_2_ States of Pure Organic Phosphors,” Journal of Materials Chemistry C 10 (2022): 14746.

[54] Q. Zhou , T. Yang , Z. Zhong , et al., “A Clustering‐Triggered Emission Strategy for Tunable Multicolor Persistent Phosphorescence,” Chemical Science 11 (2020): 2926–2933, 10.1039/C9SC06518K. 34122793 PMC8157572

[55] J. Chen , T. Yu , E. Ubba , et al., “Achieving Dual‐Emissive and Time‐Dependent Evolutive Organic Afterglow by Bridging Molecules With Weak Intermolecular Hydrogen Bonding,” Advanced Optical Materials 7 (2019): 1801593, 10.1002/adom.201801593.

[56] H. Wu , L. Gu , G. V. Baryshnikov , et al., “Molecular Phosphorescence in Polymer Matrix With Reversible Sensitivity,” ACS Applied Materials & Interfaces 12 (2020): 20765–20774, 10.1021/acsami.0c04859. 32272835

[57] B. Ding , X. Ma , and H. Tian , “Recent Advances of Pure Organic Room Temperature Phosphorescence Based on Functional Polymers,” Accounts of Materials Research 4 (2023): 827–838, 10.1021/accountsmr.3c00090.

[58] H. Ma , W. Shi , J. Ren , W. Li , Q. Peng , and Z. Shuai , “Electrostatic Interaction‐Induced Room‐Temperature Phosphorescence in Pure Organic Molecules From QM/MM Calculations,” The Journal of Physical Chemistry Letters 7 (2016): 2893–2898, 10.1021/acs.jpclett.6b01156. 27414718

[59] S. Hu , W. Zhang , C. Yao , et al., “Quasi‐Hydrogen Bond and Charge‐Trapping Effect Originating From Polar Side Group Lead to Significantly Suppressed Charge Transport in Polypropylene,” The Journal of Physical Chemistry Letters 15 (2024): 10399–10409, 10.1021/acs.jpclett.4c02619. 39383210

[60] Z. Man , Z. Lv , Z. Xu , et al., “Excitation‐Wavelength‐Dependent Organic Long‐Persistent Luminescence Originating From Excited‐State Long‐Range Proton Transfer,” Journal of the American Chemical Society 144 (2022): 12652–12660, 10.1021/jacs.2c01248. 35762534

[61] B. Ravinathan , K. Shunmugavel , S. Subramanian , et al., “Preparation and Impedance Analysis of Biodegradable Polymer Polyvinyl Alcohol With Amino Acid, Arginine,” Polymer‐Plastics Technology and Engineering 55 (2016): 889–899, 10.1080/03602559.2015.1103263.

[62] H. Yang , X. Chen , H. Lu , et al., “Self‐Trapped Excitons‐Based Warm‐White Afterglow by Room‐Temperature Engineering Toward Intelligent Multi‐Channel Information System,” Advanced Functional Materials 34 (2023): 2311437, 10.1002/adfm.202311437.

[63] C. Lin , Z. Wu , H. Ma , et al., “Charge Trapping for Controllable Persistent Luminescence in Organics,” Nature Photonics 18 (2024): 350–356, 10.1038/s41566-024-01396-0.

[64] A. C. Lazanas and M. I. Prodromidis , “Electrochemical Impedance Spectroscopy─A Tutorial,” ACS Measurement Science Au 3 (2023): 162–193, 10.1021/acsmeasuresciau.2c00070. 37360038 PMC10288619

[65] B. Peng and K. Zhang , “Confined Structural Water Molecules as Alternative Potential Emitters for Bright Photoluminescence of Thiolate‐Gold Complexes,” Chemistry—A European Journal 31 (2025): 202500499, 10.1002/chem.202500499. 39995115

[66] B. Peng , K. Zhang , and M.‐Y. He , “P‐Band Intermediate States Mediate Electron Transfer at Confined Nanoscale,” Langmuir 39 (2023): 13409–13419, 10.1021/acs.langmuir.3c01638. 37703076

[67] E. Ferrari , F. Mezzadri , and M. Masino , “Mixed‐vs‐Segregated Stack Polymorphism in the N , N , N ′, N ′‐Tetramethylbenzidine‐TCNQF 4 Charge Transfer Complex,” The Journal of Physical Chemistry C 129 (2025): 8654–8662, 10.1021/acs.jpcc.5c01376. PMC12067598 40365425

[68] N. Castagnetti , G. Kociok‐Köhn , E. Da Como , and A. Girlando , “Temperature‐Induced Valence Instability in The Charge‐Transfer Crystal TMB‐TCNQ,” Physical Review B 95 (2017): 024101, 10.1103/PhysRevB.95.024101.

[69] S. Ferrara , S. Willeit , J. P. Fuenzalida‐Werner , and R. D. Costa , “Bacterial Hybrid Light‐Emitting Diodes,” Advanced Materials 36 (2024): 2402851, 10.1002/adma.202402851. 39382232 PMC11586827

[70] Y. Atoini , L. M. Cavinato , J. Fernandez‐Cestau , Y. Gmach , D. Van Opdenbosch , and R. D. Costa , “From Blue to White: Sustainable Luminescent Metal Organic Framework for Hybrid Light‐Emitting Diodes,” Advanced Optical Materials 11 (2023): 2202643, 10.1002/adom.202202643.

[71] Y. Atoini , L. M. Cavinato , J.‐L. Schmitt , D. Van Opdenbosch , and R. D. Costa , “Stable and Efficient Rare‐Earth Free Phosphors based on an Mg( ii ) Metal–Organic Framework for Hybrid Light‐Emitting Diodes,” Dalton Transactions 53 (2024): 12455–12459, 10.1039/D4DT01690D. 39016147

[72] M. Hämmer , A. Gassmann , A. Reller , et al., “Recyclable Phosphor Sheet Based on Polyvinyl Alcohol for LED Lighting Using Remote Phosphor Technology,” Materials Technology 34 (2018): 178–183.

[73] J. D. Chaney , C. R. Goss , K. Folting , B. D. Santarsiero , and M. D. Hollingsworth , “Formyl C−H···O Hydrogen Bonding in Crystalline Bis‐Formamides?,” Journal of the American Chemical Society 118 (1996): 9432–9433, 10.1021/ja960637b.

[74] R. J. C. Brown and R. F. C. Brown , “Melting Point and Molecular Symmetry,” Journal of Chemical Education 77 (2000): 724, 10.1021/ed077p724.

