# Supplementary material for: Easily Accessible and Up‐Scalable Aliphatic Bis‐Formamides with Afterglow Luminescence: Photoluminescence Properties and Applications
Source: Small. 2026 Jun 16;22(42):e74099. doi: 10.1002/smll.74099 (PMC13410431; doi:10.1002/smll.74099)
Supplement: Supplementary file 1 — Supporting File: smll74099‐sup‐0001‐SuppMat.pdf. [file SMLL-22-e74099-s001.pdf]

Supporting Information

Easily Accessible and Up-Scalable Aliphatic Bis-Formamides with Afterglow Luminescence: Photoluminescence Properties and Applications

Chengyi Zhu,[a, b] Sara Ferrara,[c] Anna Zieleniewska,[c] Daniel Van Opdenbosch,[d] Rubén D. Costa,[c] Youssef Atoini,[c]* and Jean-Marie Lehn[a, b]*

[a] Dr. Chengyi Zhu, Prof. Dr. Jean-Marie Lehn
Lehn Institute of Functional Materials, School of Chemistry,
Sun Yat-Sen University, 510275 Guangzhou, China
[b] Dr. Chengyi Zhu, Prof. Dr. Jean-Marie Lehn
Laboratoire de Chimie Supramoléculaire,
Institut de Science et d'Ingénierie Supramoléculaires,
University of Strasbourg,
8 Allée Gaspard Monge, 67000 Strasbourg, France.
E-mail: lehn@unistra.fr
[c] Dr. Sara Ferrara, Dr. Anna Zieleniewska, Prof. Dr. Rubén D. Costa, Dr. Youssef Atoini
Technical University of Munich,
Campus Straubing for Biotechnology and Sustainability,
Chair of Biogenic Functional Materials,
Schulgasse, 22, Straubing 94315, Germany,
E-mail: y.atoini@tum.de
[d] Dr. Daniel Van Opdenbosch
Technical University of Munich, Campus Straubing for Biotechnology and Sustainability, Chair for Biogenic Polymers, Schulgasse 16, Straubing 94315, Germany.

Contents

Materials and Measurements. **3**

Synthesis and Characterization of BFAC6: **4**

Preparation of BFAC6@PVA films for decoration and anti-counterfeiting applications: 7

Preparation of TMB-BFAC6: 7

Preparation of the photon down-converting coatings, TMB-BFAC6@PVA: 8

Single crystal data of BFAC6: 8

Characterization of BFACn compounds: 14

Materials and Measurements.

Nuclear magnetic resonance (NMR) data were collected on Bruker AVANCE III 400 (400 MHz) Spectrometer and Bruker AVANCE III 500 (500 MHz).

High resolution mass spectrometry (HRMS) were obtained on ThermoFisher Exactive Plus EMR Orbitrap mass spectrometer using electrospray ionization technique.

Infrared (IR) experiments were performed using SHIMADZU IRAffinity-1S Fourier transform infrared spectrophotometer in the region of 4000-700 cm^{-1}.

Elemental analyses (C, H and N) were performed on a Thermo Scientific FLASH 2000 CHNS/O Analyzer.

Melting points were measured on a digital melting point apparatus from Büchi, Model B-540 after a calibration certification with standards.

Single-crystal X-ray diffraction data for crystals were collected on a Rigaku Oxford SuperNova X-RAY diffractometer system equipped with a Mo sealed tube (λ = 0.71073 Å). The structure was solved and refined using the Bruker SHELXTL Software Package. All hydrogen atoms were located in calculated positions and refined anisotropically. The single crystal data have been deposited in the Cambridge Crystallographic Data Center

CCDC No. of BFAC2： 2479052, BFAC4： 2479053, BFAC6： 2150359, BFAC7： 2099953, BFAC8： 2150358, BFAC10： 2099962, BFAC12： 2099963

Photoluminescence pictures were taken under a UV lamp using UV radiation of 370 nm. Excitation/emission spectra and time-resolved measurements were recorded with a HORIBA FluoroLog-3 and an Edinburgh FS5 spectrometers. A pulsed xenon lamp was used to excite the samples for the steady-state measurements, and a photodiode was used for the time-resolved measurements. The photoluminescence quantum yields were measured with an absolute photoluminescence quantum yield measurement integrating sphere (Hamamatsu, C11347-11).

The impedance measurements were carried out using a potentiostat PGSTAT204, Metrohm Autolab with FRA32M module for impedance analysis. The PVA and BFAC6 films were placed between two FTO electrodes. A frequency range of 0.01 Hz–150 kHz was used with an applied ac bias of 100 mV (no dc bias was applied). The system was illuminated using 370 nm LED irradiation of 50 mW cm^{-2} (WINGER Power LED Star) as pumping sources. The fit and simulation were done using Nova 2.1.5 software.

The HLEDs were fabricated by placing the 380 nm down-converting coating on top of an LED chip (380 nm, WINGER Power LED Star) with irradiation ranging between 10 and 200 mA,

200 mA corresponding to a photon flux of 80 mW cm^{-2}.

Synthesis and Characterization of BFAC6:

BFAC6 was prepared from diamino hexane and ethyl formamide following the reported procedures.[S1-S3]

Yield: 91%. Melting point: 107.4-108.5 °C. ^{1}H NMR (500 MHz, DMSO-*d6*, 25 °C, TMS): δ = 7.98(s, CHO), 7.91(s, CHO), 3.07(m, 4H, CH2), 1.39(m, 4H, CH2), 1.25(m, 4H, CH2). ^{13}C - NMR (126 MHz, D_2O, 25 °C, TMS): δ = 164.93, 161.34, 41.21, 37.44, 31.29, 29.40, 26.45, 26.39, 25.97. HRMS (ESI$^+$, MeOH, *m/z*): calcd for $[M + H]^+$, 173.127; found, 173.128. Anal. Calcd for (C8H16N2O2): C, 55.79; H, 9.36; N, 16.27. Found: C, 55.79; H, 9.38; N, 16.22.

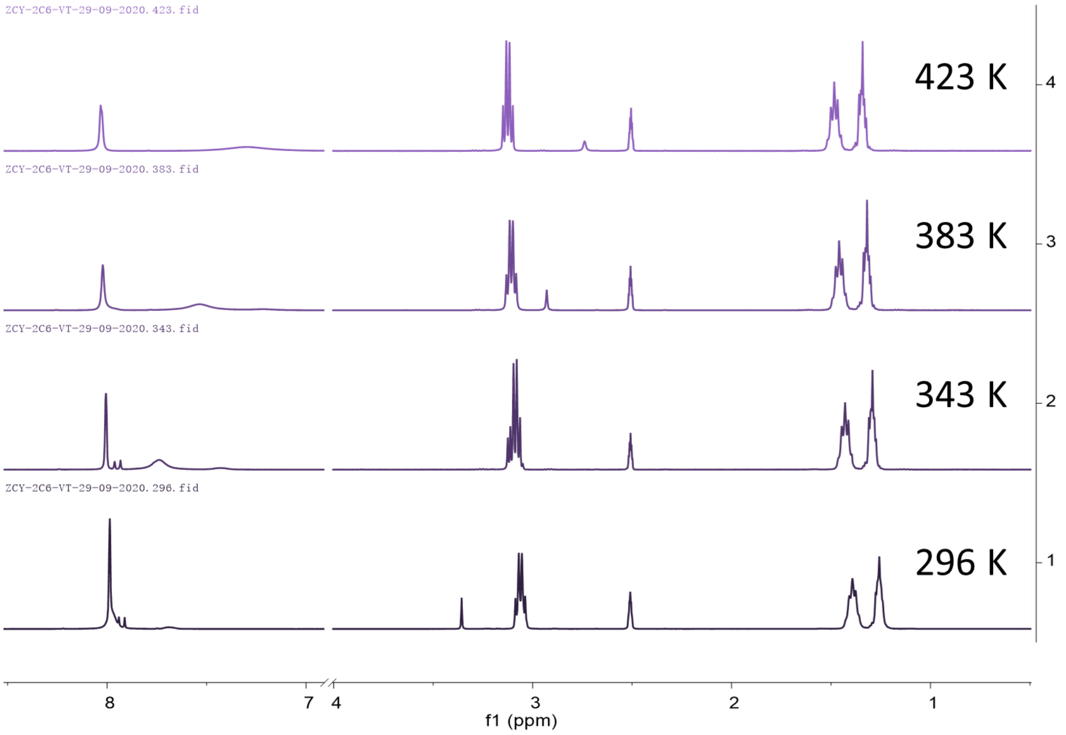

Figure S1. ^{1}H NMR spectra of **BFAC6** in DMSO-d6 at different temperatures.

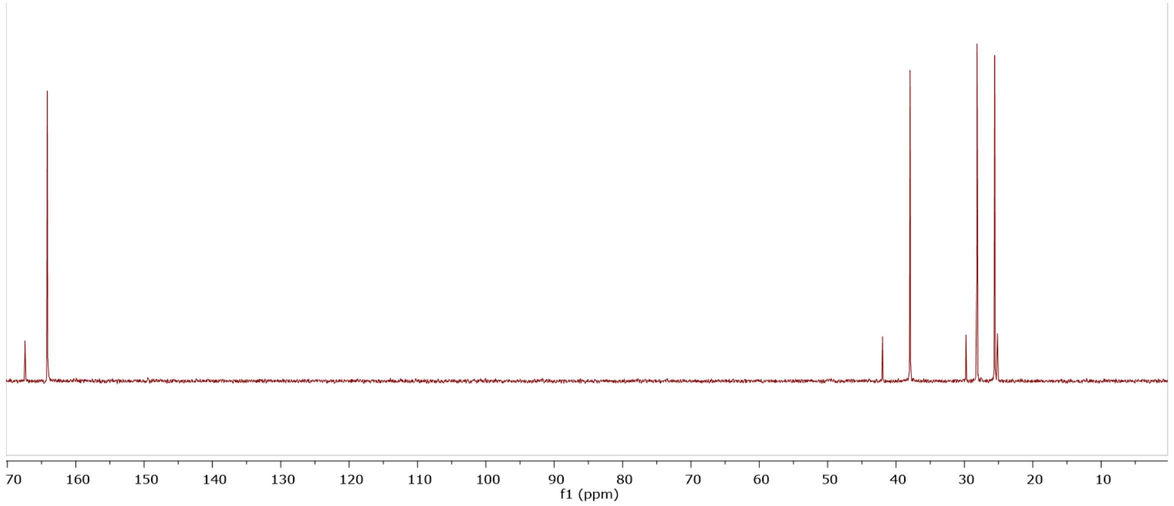

Figure S2. ^{13}C NMR spectrum of **BFAC6** in D_2O at 293 K.

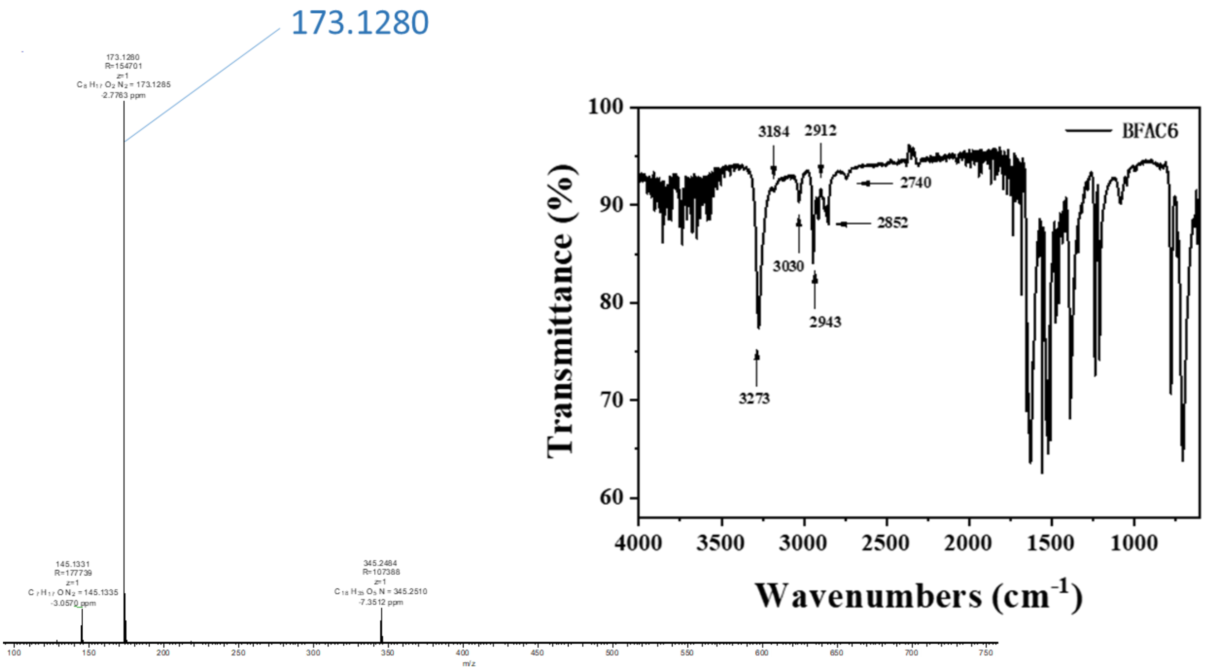

Figure S3. ESI-HRMS spectrum and IR spectrum of **BFAC6**.

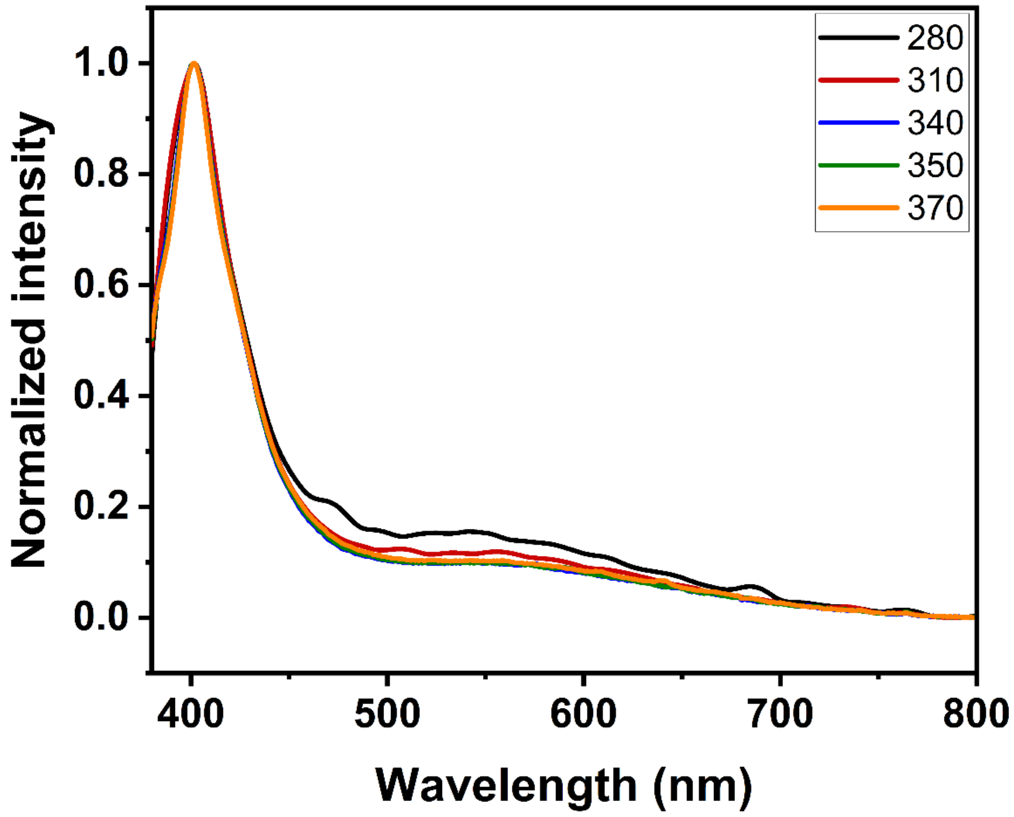

Figure S4. Emission spectra of **BFAC6** at different excitation wavelengths – see legend, values in nm.

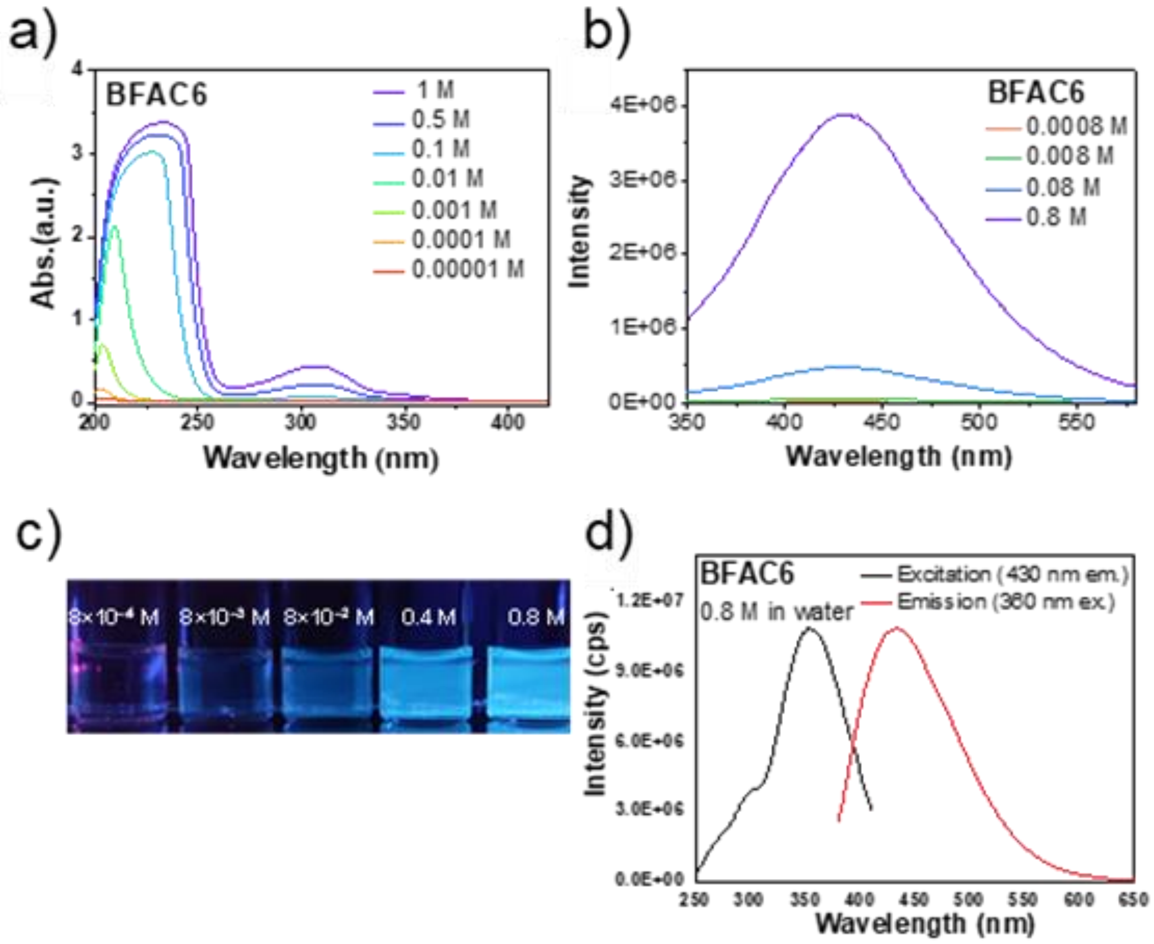

Figure S5. a) UV-Vis absorption spectra of **BFAC6** aqueous solutions with different concentration; b) emission spectra of aqueous solutions of **BFAC6** at different concentrations; c) photographs of **BFAC6** aqueous solutions at different concentration taken under 365 nm UV lamp; d) excitation and emission spectra of a 0.8 M **BFAC6** aqueous solution.

Preparation of BFAC6@PVA films for decoration and anti-counterfeiting applications:

BFAC6 is dispersed in 5% PVA (polyvinyl alcohol, 5g in 100 mL deionized water (50 mg/mL)) aqueous solution with certain content (70 mg/mL, 0.4 M), then a homogeneous solution was obtained after ultrasonication for 20 min. The solution was injected into a pen cartridge to yield security ink for painting.

The obtained **BFAC6@PVA** homogeneous solution is painted on glass surface, dried naturally to form the film, then dried under 60 ºC in a vacuum oven overnight.

Preparation of TMB-BFAC6:

TMB-BFAC6 was obtained through a typical melt-casting method in which the mixture of two compounds (10mg of TMB for 1g of BFAC6) were heated over the melting point of BFAC6 (150°C) under a nitrogen atmosphere and then cooled slow to room temperature at different

rates: i) slow cooling of 10°C steps to rt, regular cooling to rt by stopping the heating, iii) sudden cooling to -78°C, iv) sudden cooling to -196°C.

Preparation of the photon down-converting coatings, TMB-BFAC6@PVA:

A 80 mg·mL^{-1} polyvinyl alcohol (PVA, average Mw= 146,000 – 186,000 g/mol) solution in water was made by mixing 500 mg PVA in 10 mL of water. prior to heating at 80°C and stirring overnight. 70 mg of the emissive material, **TMB-BFAC6** was put in a vial and 900 µL of the PVA solution in water was poured in the vial. The resulting suspension was heated at 80°C and stirred at 300 rpm for 30 minutes, until the powder is fully dissolved. The final suspension was allowed to cool down to r. t., and 300 µL of it were put in a well-shaped Teflon mold. The suspension was left to dry for 48h at ambient conditions, and the resulting dry, dome-shaped coating was recovered. The thickness of the coating was measured using a Helios-Preisser electronic outside micrometer (thickness measured: 1.49 ±0.05 mm).

Single crystal data of BFAC6:

Table S1. Crystal data and structure refinement for **BFAC6.**

Empirical formula	$C_8H_{16}N_2O_2$
Formula weight	172.23
Crystal system	monoclinic
Space group	$P2_1/n$
a/Å	8.1013(5)
b/Å	4.6433(2)
c/Å	12.2119(7)
α/°	90
β/°	95.459(2)
γ/°	90
Volume/Å^3	457.29(4)
Z	2
Calculated density	1.251
F(000)	188.0
Radiation	MoKα (λ = 0.71073)
2 theta range for data collection	5.79 to 56.006
Limiting indices	$-10 \leq h \leq 10$, $-6 \leq k \leq 5$, $-16 \leq l \leq 16$
Reflections collected	17081
Independent reflections	1111 [R_{int} = 0.0485, R_{sigma} = 0.0178]
Data/restraints/parameters	1111/0/59
Quality-of-fit indicator	1.034

Final *R* indices [*I*>2σ(*I*)]	R_1 = 0.0371, wR_2 = 0.0890
R indices (all data)	R_1 = 0.0472, wR_2 = 0.0972
Largest diff. peak and hole	0.26/-0.18

Table S2. Selected bond distances for **BFAC6**.

Atom 1	Atom 2	Bond distances (Å)
**C1**	O1	1.2338(16)
**C1**	N1	1.3295(15)
**C2**	N1	1.4622(13)

Table S3. Selected bond angles for **BFAC6.**

Atom 1	Atom 2	Atom 3	Angle (°)
O1	C1	N1	124.28(11)
N1	C2	C3	111.95(10)
C1	N1	C2	121.08(10)

Table S4. Hydrogen bonds in **BFAC6** packing.

D	H	A	d(D-H) (Å)	d(H-A) (Å)	d(D-A) (Å)	D-H-A (°)
N(1)	H(1N)	$O(1)^3$	0.875(17)	2.030(17)	2.8678(13)	160.1(14)

Symmetry transformations used to generate equivalent atoms:

^{1}1/2-X,1-Y,1/2+Z, ^{2}1-X,1-Y,1-Z, ^{3}+X,1+Y,+Z, ^{4}-1/2+X,1/2-Y,-1/2+Z, ^{5}-1+X,+Y,+Z, ^{6}1/2-X,-1/2+Y,3/2-Z, ^{7}1+X,+Y,+Z

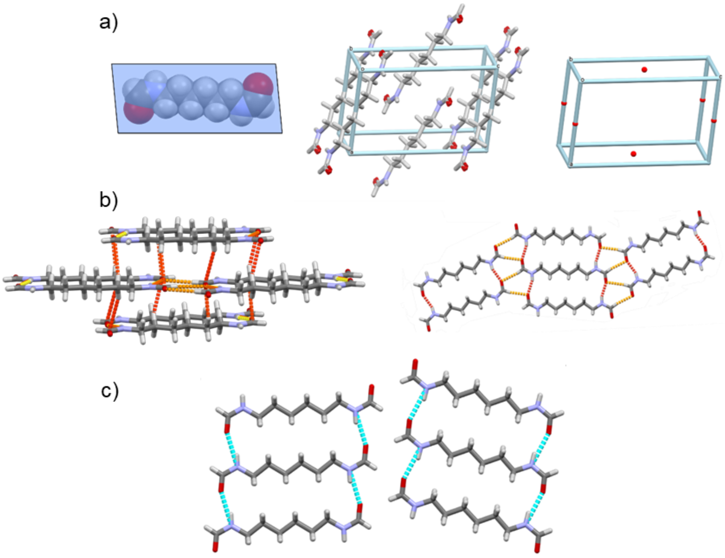

Figure S6. a) The molecular structure of **BFAC6** and in its corresponding monoclinic unit cell. b) Interlayer structure of **BFAC6**. c) Intermolecular hydrogen bonds N-H···O of **BFAC6**.

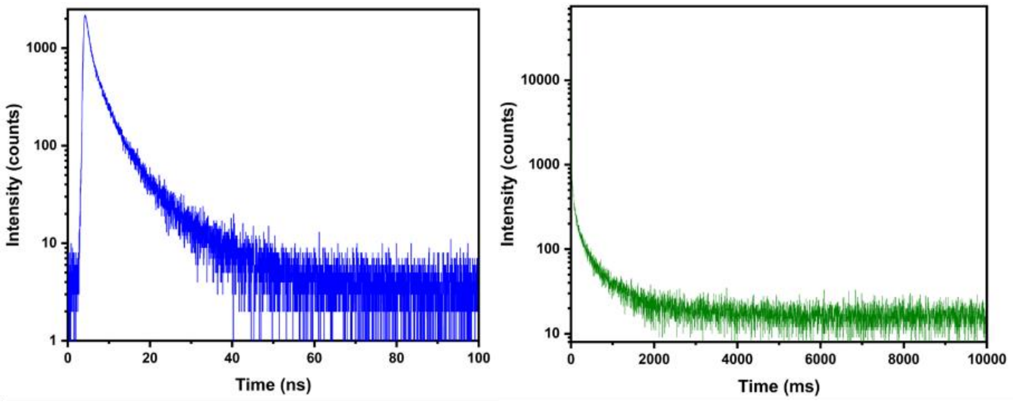

Figure S7. Decay plot at 420 nm at 375 nm excitation (left); phosphorescence decay plot at 550 nm) (right) of **BFAC6@PVA**.

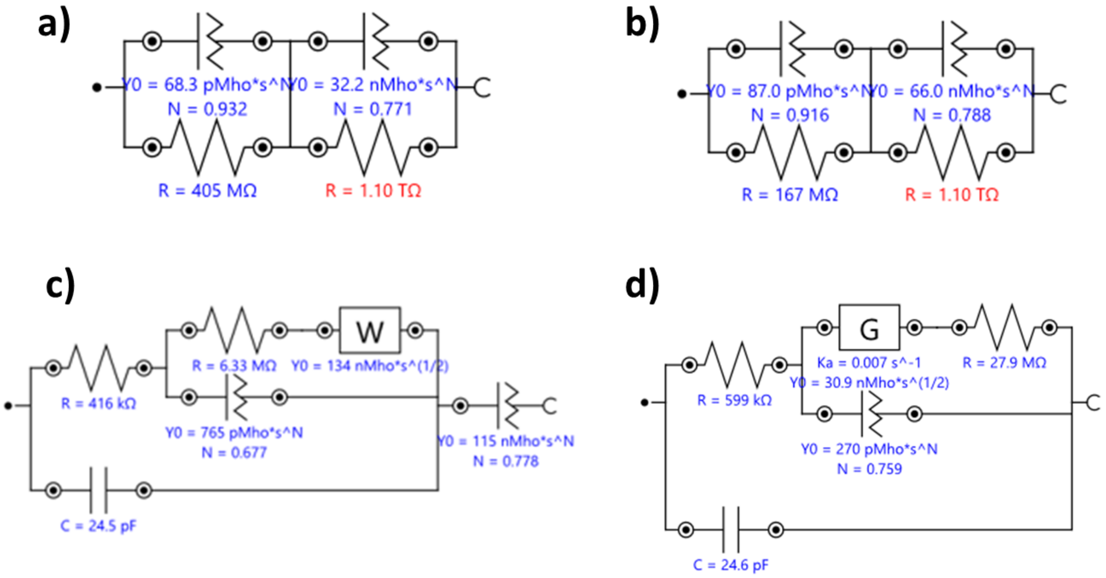

Figure S8. Equivalent electric circuit schemes used for the fitting of Nyquist and Phase angle plots of PVA in dark (a) and upon illumination (b), and **BFAC6@PVA** in dark (c) and upon 370 nm illumination (d) presented in **Figure 4**. The fitted values are presented on the circuit schemes. Convergence required fixing one parameter (shown in red). Allowing this parameter to vary does not affect the extracted resistance of the first semicircle.

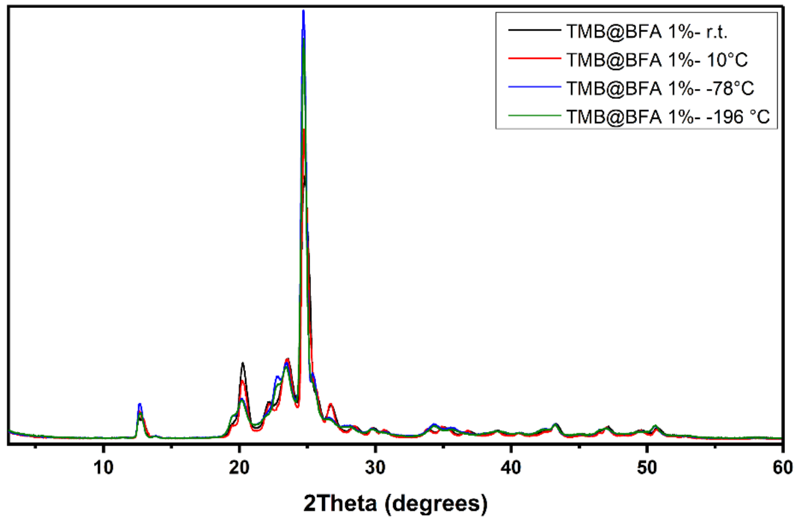

Figure S9. PXRD patterns of **TMB-BFAC6** upon different crystallization processes.

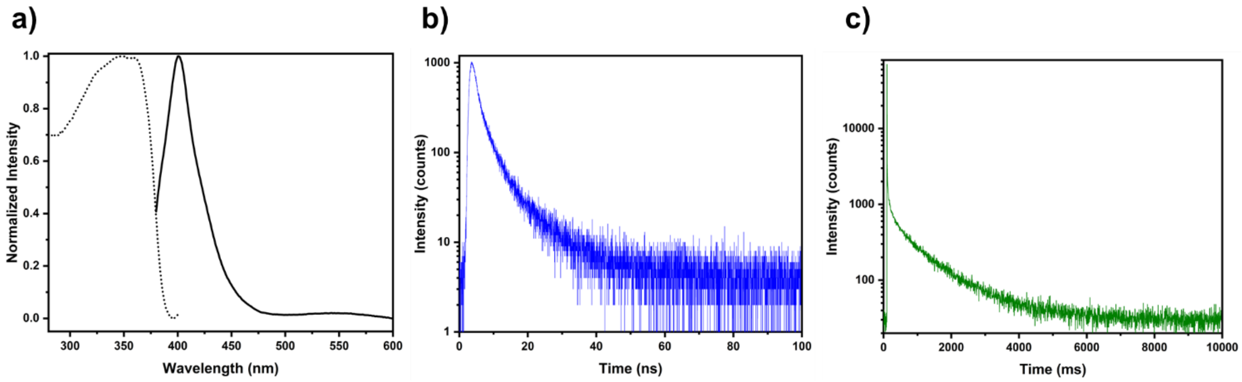

Figure S10. Excitation (dotted line, $\lambda_{Em} = 420$ nm) and emission (full line, $\lambda_{Exc} = 370$ nm) (a); fluorescence decay plot ($\lambda_{Em} = 420$ nm) (b); phosphorescence decay plot, ($\lambda_{Em} = 550$ nm) (c) of **TMB-BFAC6** in crystalline powder state.

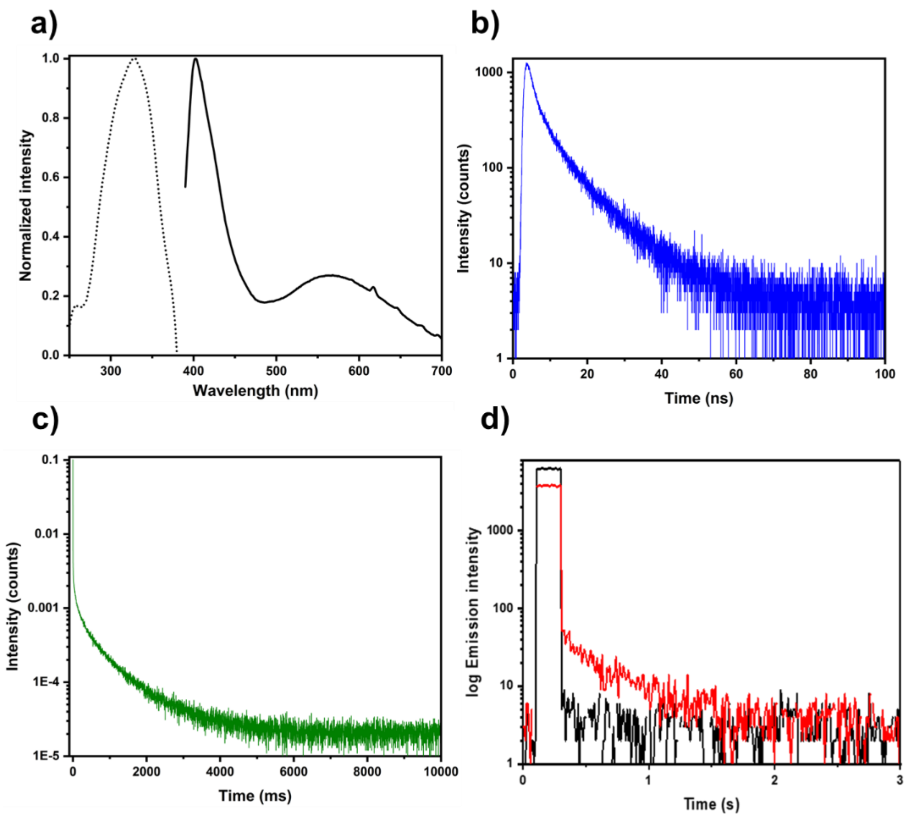

Figure S11. Photophysical properties of **TMB-BFAC6@PVA**. (a) Excitation (dotted line, λ_{Em} = 420 nm) and emission (full line, λ_{Exc} = 370 nm); (b) Prompt emission decay plot (λ_{Em} = 420 nm); (c) Long-lived emission decay plot, (λ_{Em} = 550 nm); (d) Kinetics scans of **TMB-BFAC6@PVA** (black line: λ_{Em}=420 nm; red line: λ_{Em}=550 nm).

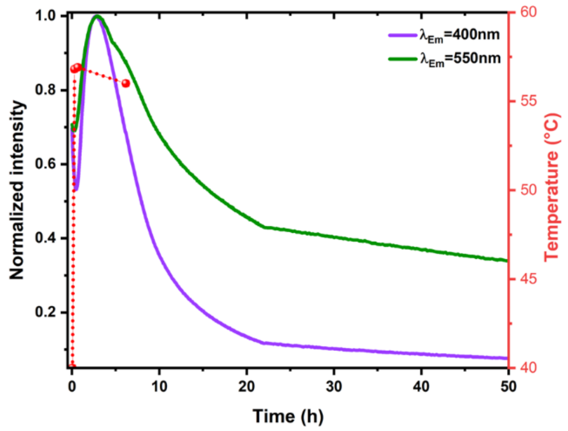

Figure S12. Left: Intensity decay profile of **TMB-BFAC6@PVA** high (λ_{Em}=400 nm, violet) and low (and λ_{Em} = 550 nm, green) energy emission bands over time upon operating the device at 200 mA.

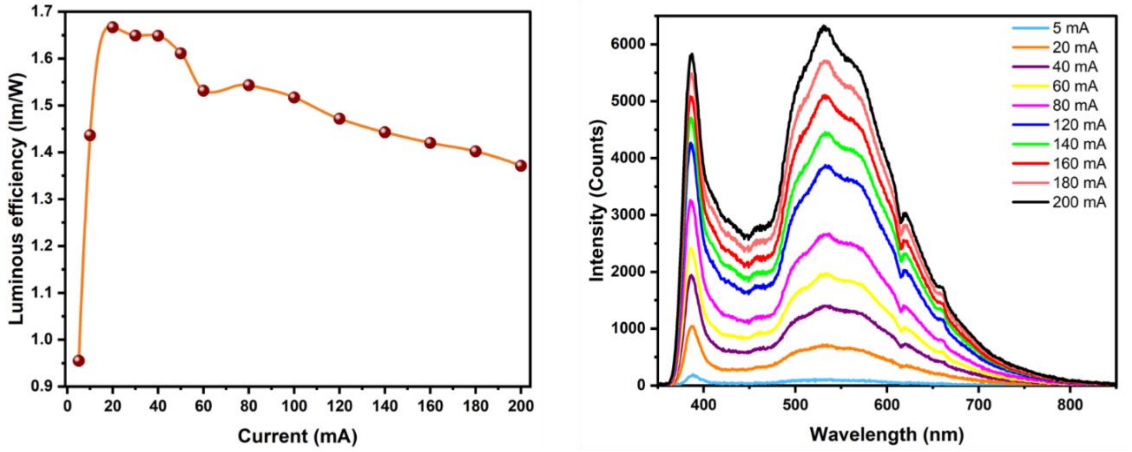

Figure S13. Left: Luminous efficiency of the device at different applied currents in a range 10-200 mA; Right: Emission spectra of the **TMB-BFAC6@PVA** device at different applied currents – see legend. All the data were acquired in ambient conditions.

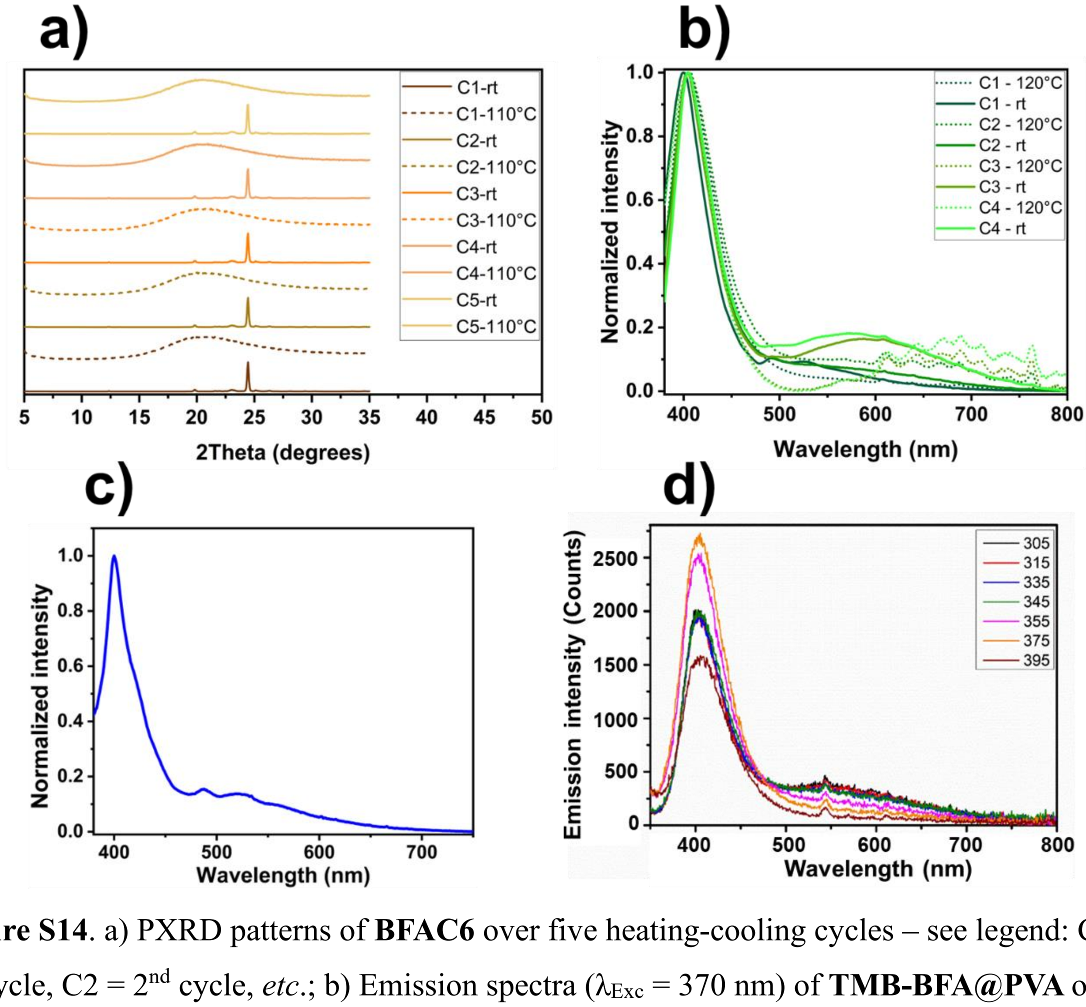

Figure S14. a) PXRD patterns of **BFAC6** over five heating-cooling cycles – see legend: C1 = 1st cycle, C2 = 2nd cycle, *etc*.; b) Emission spectra (λ_{Exc} = 370 nm) of **TMB-BFA@PVA** over four heating-cooling cycles – see legend: C1 = 1st cycle, C2 = 2nd cycle, *etc*.; c) Emission spectrum (λ_{Exc} = 370 nm) of BFAC6 at 77 K; d) Emission spectra of **TMB-BFAC6@PVA** coatings at upon increasing temperature in a 305-395 K range – see legend.

Characterization of BFACn compounds:

BFAC2: Yield: 88 %. Melting point: 107.0-108.2 °C (ref. 105 °C). ^{1}H NMR (500 MHz, DMSO-*d6*, 25 °C, TMS): δ = 3.15(m, 4H, methylene CH2), 7.67(s, NH), 7.86(s, CHO), 8.01(s, CHO), 8.04(s, NH). ^{13}C -NMR (126 MHz, DMSO-*d6*, 25 °C, TMS): δ = 37.32, 161.85, 165.09. HRMS (ESI^+, MeOH, *m/z*): calcd for $[M + H]^+$, 117.066; found, 117.066. Anal. Calcd for (C4H8N2O2): C, 41.37; H, 6.94; N, 24.13. Found: C, 41.36; H, 6.95; N, 23.97.

BFAC4: Yield: 86 %. Melting point: 92.8-94.1 °C (ref. 92.5 °C). ^{1}H NMR (500 MHz, DMSO-*d6*, 25 °C, TMS): δ = 7.99(s, CHO), 7.91(s, CHO), 3.07(m, 4H, CH2), 1.40(m, 4H, CH2). ^{13}C -NMR (126 MHz, DMSO-*d6*, 25 °C, TMS): δ = 164.97, 161.41, 40.93, 37.18, 37.10, 26.93. HRMS (ESI^+, MeOH, *m/z*): calcd for $[M + H]^+$, 145.097; found, 145.097. Anal. Calcd for (C6H12N2O2): C, 49.99; H, 8.39; N, 19.43. Found: C, 49.97; H, 8.36; N, 19.35.

BFAC5: Yield: 75 %. Melting point: 61.3-62.7 °C (ref. 64.5 °C). ^{1}H NMR (500 MHz, DMSO-*d6*, 25 °C, TMS): δ = 7.99(s, CHO), 7.91(s, CHO), 3.05(m, 4H, CH2), 1.40(m, 4H, CH2), 1.25(m, 2H, CH2). ^{13}C -NMR (126 MHz, DMSO-*d6*, 25 °C, TMS): δ = 164.93, 161.37, 41.19, 37.42, 30.96, 29.08, 24.14, 23.68. HRMS (ESI^+, MeOH, *m/z*): calcd for $[M + H]^+$, 159.112; found, 159.112. Anal. Calcd for (C7H14N2O2): C, 53.15; H, 8.92; N, 17.71. Found: C, 52.13; H, 8.79; N, 17.35.

BFAC7: Yield: 90 %. Melting point: 73.6-75.8 °C (ref. 77.5 °C). ^{1}H NMR (500 MHz, DMSO-*d6*, 25 °C, TMS): δ = 7.98(s, CHO), 7.91(s, CHO), 3.05(m, 4H, CH2), 1.39(m, 4H, CH2), 1.25(m, 6H, CH2). ^{13}C -NMR (126 MHz, DMSO-*d6*, 25 °C, TMS): δ = 164.92, 161.33, 41.26, 37.48, 31.29, 29.41, 28.79, 28.72, 26.76, 26.27. HRMS (ESI^+, MeOH, *m/z*): calcd for $[M + H]^+$, 187.144; found, 187.144. Anal. Calcd for (C9H18N2O2): C, 58.04; H, 9.74; N, 15.04. Found: C, 57.85; H, 9.73; N, 14.90.

BFAC8: Yield: 85 %. Melting point: 91.6-92.8 °C (ref. 87 °C). ^{1}H NMR (500 MHz, DMSO-*d6*, 25 °C, TMS): δ = 7.98(s, CHO), 7.91(s, CHO), 3.06(m, 4H, CH2), 1.39(m, 4H, CH2), 1.25(m, 8H, CH2). ^{13}C -NMR (126 MHz, DMSO-*d6*, 25 °C, TMS): δ = 164.91, 161.32, 41.28, 37.49, 31.34, 29.44, 29.09, 29.04, 26.75, 26.28. HRMS (ESI^+, MeOH, *m/z*): calcd for $[M + H]^+$, 201.158; found, 201.159. Anal. Calcd for (C10H20N2O2): C, 59.97; H, 10.07; N, 13.99. Found: C, 59.98; H, 10.14; N, 13.99.

BFAC10: Yield: 78 %. Melting point: 97.9-99.1 °C. ^{1}H NMR (500 MHz, DMSO-*d6*, 25 °C, TMS): δ = 7.98(s, CHO), 7.91(s, CHO), 3.06(m, 4H, CH2), 1.40(m, 4H, CH2), 1.25(m, 12H, CH2). ^{13}C -NMR (126 MHz, DMSO-*d6*, 25 °C, TMS): δ = 164.89, 161.30, 41.28, 37.49, 31.37, 29.47, 29.41, 29.39, 29.15, 29.09, 26.81, 26.33. HRMS (ESI^+, MeOH, *m/z*): calcd for $[M + H]^+$,

229.191; found, 229.190. Anal. Calcd for (C12H24N2O2): C, 63.12; H, 10.59; N, 12.27. Found: C, 63.14; H, 10.67; N, 12.30.

BFAC12: Yield: 65 %. Melting point: 102.7-104.9 °C. ^{1}H NMR (500 MHz, CDCl3, 25 °C, TMS): δ = 8.10(s, CHO), 7.96(s, CHO), 3.23(m, 4H, CH2), 1.45(m, 4H, CH2), 1.19(m, 16H, CH2). ^{13}C -NMR (126 MHz, CDCl3, 25 °C, TMS): δ = 164.62, 161.20, 41.76, 38.20, 31.21, 29.52, 29.50, 29.43, 29.41, 29.39, 29.17, 29.13, 29.08, 26.80, 26.77, 26.33. HRMS (ESI$^+$, MeOH, *m/z*): calcd for [M + H]$^+$, 257.222; found, 257.221. Anal. Calcd for (C14H28N2O2): C, 65.59; H, 11.01; N, 10.93. Found: C, 65.60; H, 11.02; N, 10.83.

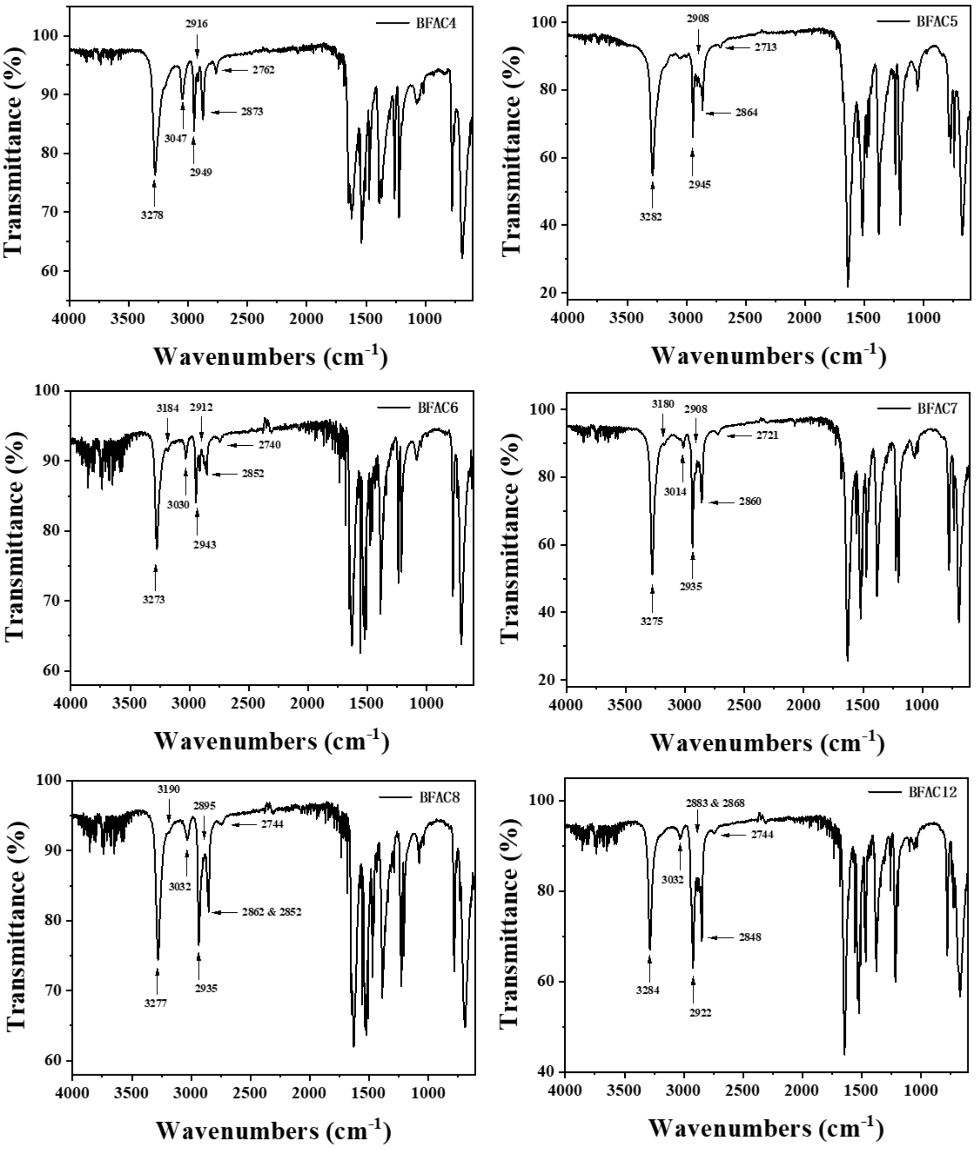

Figure S15. IR spectra of **BFACn** (n = 4, 5, 6, 7, 8, 12) in solid state.

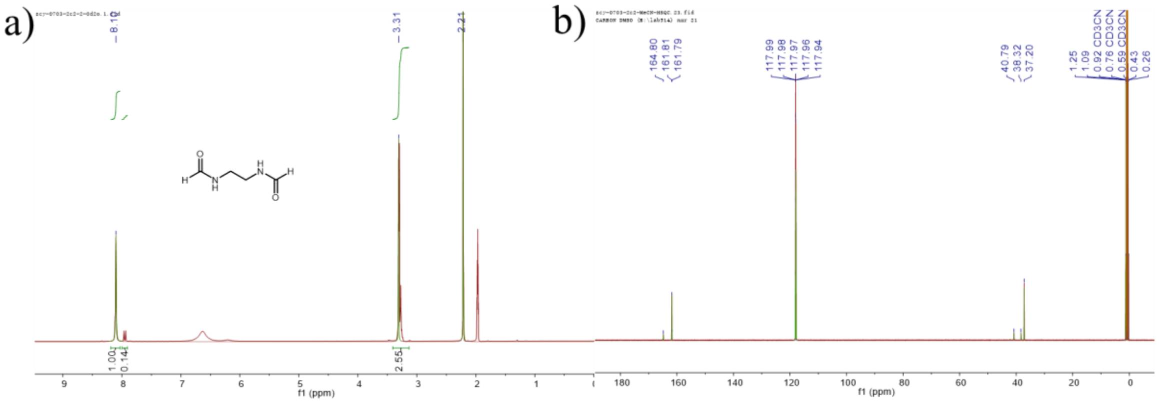

Figure S16. a) the liquid state ^{1}H NMR of **BFAC2** in methanol-d4, b) the liquid state ^{13}C NMR of **BFAC2** in DMSO-D6.

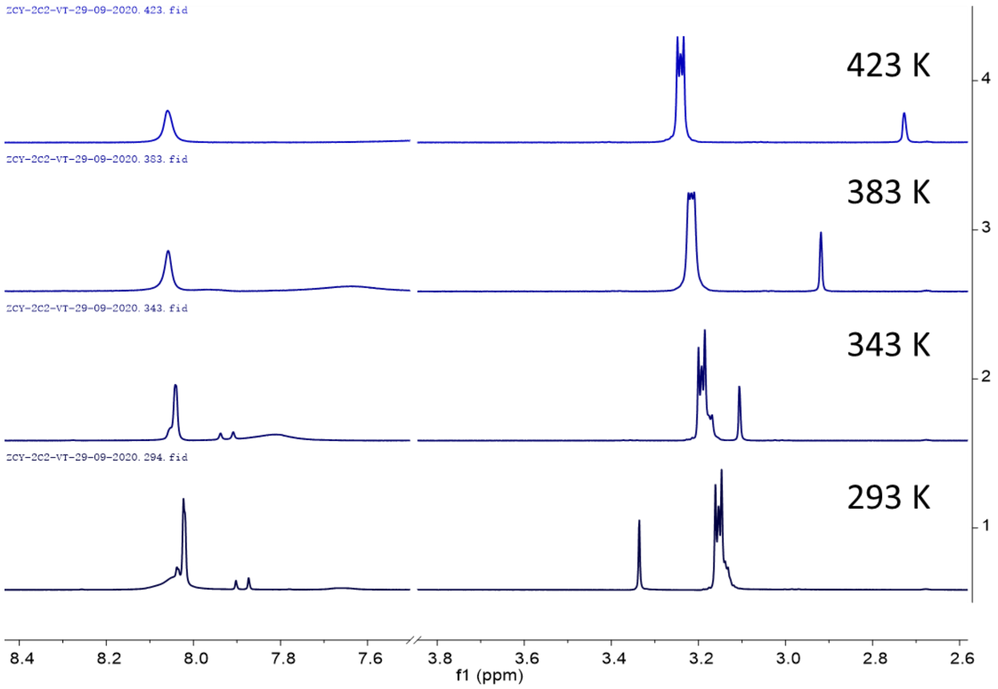

Figure S17. ^{1}H NMR spectrum of **BFAC2** in DMSO-D6 at different temperature.

Table S5. Crystal data and structure refinement for **BFAC2**.

Empirical formula	$C_4H_8N_2O_2$
Formula weight	116.12
Crystal system	orthorhombic
Space group	Pbca
a/Å	8.7478(4)
b/Å	6.6932(3)
c/Å	9.3619(4)
α/°	90

β/°	90
γ/°	90
Volume/Å^3	548.15(4)
Z	4
Calculated density	1.407
F(000)	248.0
Radiation	MoKα (λ = 0.71073)
2 theta range for data collection	8.708 to 64.094
Limiting indices	-13 ≤ h ≤ 13, -9 ≤ k ≤ 9, -13 ≤ l ≤ 13
Reflections collected	14308
Independent reflections	942 [R_{int} = 0.0184, R_{sigma} = 0.0103]
Data/restraints/parameters	942/0/41
Quality-of-fit indicator	1.101
Final *R* indices [I>2σ(I)]	R_1 = 0.0285, wR_2 = 0.0838
R indices (all data)	R_1 = 0.0293, wR_2 = 0.0848
Largest diff. peak and hole	0.39/-0.20

Table S6. Selected bond distances for **BFAC2**.

Atom 1	Atom 2	Bond distances (Å)
**C(1)**	O(1)	1.2401(8)
**C(1)**	N(1)	1.3310(8)
**C(2)**	N(1)	1.4550(8)
**C(2)**	C(2)^1	1.5307(12)

Table S7. Selected bond angles for **BFAC2**.

Atom 1	Atom 2	Atom 3	Angle (°)
O(1)	C(1)	N(1)	124.34(6)
N(1)	C(2)	C(2)^1	110.94(6)
C(1)	N(1)	C(2)	121.97(5)

Symmetry transformations used to generate equivalent atoms:

^{1}1-X,1-Y,1-Z

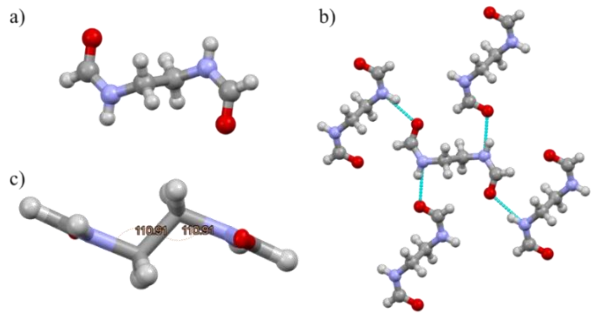

Figure S18. a) and c) The crystal structure of **BFAC2**, different views b) The intermolecular hydrogen bonding of **BFAC2**.

Table S8. Crystal data and structure refinement for **BFAC4**.

Empirical formula	$C_6H_{12}N_2O_2$
Formula weight	144.18
Crystal system	Monoclinic
Space group	$P2_1/n$
a/Å	5.6495(6)
b/Å	4.6887(5)
c/Å	13.6473(15)
α/°	90
β/°	96.620(4)
γ/°	90
Volume/Å^3	359.09(7)
Z	2
Calculated density	1.333
F(000)	156.0
Radiation	MoKα (λ = 0.71073)
2 theta range for data collection	6.01 to 55.87
Limiting indices	$-7 \leq h \leq 7$, $-6 \leq k \leq 5$, $-17 \leq l \leq 18$
Reflections collected	9413
Independent reflections	863 [R_{int} = 0.0572, R_{sigma} = 0.0263]
Data/restraints/parameters	863/0/50
Quality-of-fit indicator	1.057
Final *R* indices [I>2σ(I)]	R_1 = 0.0389, wR_2 = 0.0970
R indices (all data)	R_1 = 0.0455, wR_2 = 0.1033
Largest diff. peak and hole	0.22/-0.20

Table S9. Selected bond distances for **BFAC4**.

Atom 1	Atom 2	Bond distances (Å)
**C(1)**	O(1)	1.2336(16)
**C(1)**	N(1)	1.3261(14)
**C(2)**	N(1)	1.4630(13)
**C(2)**	C(3)	1.5202(15)
**C(3)**	C(3)[1]	1.531(2)

Table S10. Selected bond angles for **BFAC4**.

Atom 1	Atom 2	Atom 3	Angle (°)
O(1)	C(1)	N(1)	124.83(10)
N(1)	C(2)	C(3)	111.04(9)
C(2)	C(3)	C(3)[1]	110.94(11)
C(1)	N(1)	C(2)	121.39(10)

Symmetry transformations used to generate equivalent atoms:

[1]-X,1-Y,1-Z

Table S11. Crystal data and structure refinement for **BFAC7**.

Empirical formula	$C_9H_{18}N_2O_2$
Formula weight	186.25
Crystal system	monoclinic
Space group	C2/c
a/Å	14.0351(5)
b/Å	4.5728(2)
c/Å	16.5054(6)
α/°	90
β/°	110.0330(10)
γ/°	90
Volume/Å^3	995.22(7)
Z	4
Calculated density	1.243
F(000)	408.0
Radiation	MoKα (λ = 0.71073)
2 theta range for data collection	5.254 to 59.984
Limiting indices	$-19 \leq h \leq 19$, $-6 \leq k \leq 6$, $-23 \leq l \leq 23$
Reflections collected	15401
Independent reflections	1458 [R_{int} = 0.0274, R_{sigma} = 0.0136]
Data/restraints/parameters	1458/0/68
Quality-of-fit indicator	1.075
Final *R* indices [*I*>2σ(*I*)]	R_1 = 0.0316, wR_2 = 0.0901
R indices (all data)	R_1 = 0.0363, wR_2 = 0.0950
Largest diff. peak and hole	0.35/-0.14

Table S12. Selected bond distances (Å) for **BFAC7**

**.**Atom 1	**Atom 2**	**Bond distances (Å)**
C1	O1	1.2348(10)
C1	N1	1.3316(9)
C2	N1	1.4588(9)

Table S13. Selected bond angles (deg) for **BFAC7**.

**Atom 1**	**Atom 2**	**Atom 3**	**Angle(º)**
O1	C1	N1	124.01(7)
N1	C2	C3	113.17(6)
C1	N1	C2	120.58(6)

Table S14. Crystal data and structure refinement for **BFAC8**.

Empirical formula	$C_{10}H_{20}N_2O_2$
Formula weight	200.28
Crystal system	monoclinic
Space group	$P2_1/c$
a/Å	7.8234(3)
b/Å	4.6838(2)
c/Å	15.1162(6)
α/°	90
β/°	96.739(2)
γ/°	90
Volume/Å^3	550.08(4)
Z	2
Calculated density	1.209
F(000)	220.0
Radiation	MoKα (λ = 0.71073)
2 theta range for data collection	5.244 to 58.286
Limiting indices	$-10 \leq h \leq 10$, $-6 \leq k \leq 6$, $-20 \leq l \leq 20$
Reflections collected	21020
Independent reflections	1485 [R_{int} = 0.0188, R_{sigma} = 0.0098]

Data/restraints/parameters	1485/0/68
Quality-of-fit indicator	1.147
Final *R* indices [I>2σ(I)]	R_1 = 0.0298, wR_2 = 0.0867
R indices (all data)	R_1 = 0.0310, wR_2 = 0.0879
Largest diff. peak and hole	0.34/-0.19

Table S15. Selected bond distances for **BFAC8**.

Atom 1	**Atom 2**	**Bond distances (Å)**
C1	O1	1.2353(10)
C1	N1	1.3307(9)
C2	N1	1.4589(9)

Table S16. Selected bond angles (deg) for **BFAC8**.

Atom 1	Atom 2	Atom 3	Angle/°
O1	C1	N1	124.11(7)
N1	C2	C3	112.21(6)
C1	N1	C2	120.86(6)

Table S17. Crystal data and structure refinement for **BFAC10**.

Empirical formula	$C_{12}H_{24}N_2O_2$
Formula weight	228.33
Crystal system	triclinic
Space group	P-1
a/Å	4.6712(2)
b/Å	5.4857(2)
c/Å	12.4311(5)
α/°	82.6190(10)
β/°	86.0510(10)
γ/°	84.7860(10)
Volume/Å^3	314.07(2)
Z	1
Calculated density	1.207
F(000)	126.0
Radiation	MoKα (λ = 0.71073)

2 theta range for data collection	6.622 to 58.33
Limiting indices	$-6 \leq h \leq 6$, $-7 \leq k \leq 7$, $-17 \leq l \leq 16$
Reflections collected	24250
Independent reflections	1695 [R_{int} = 0.0234, R_{sigma} = 0.0097]
Data/restraints/parameters	1695/0/77
Quality-of-fit indicator	1.138
Final *R* indices [$I>2\sigma(I)$]	R_1 = 0.0302, wR_2 = 0.0910
R indices (all data)	R_1 = 0.0336, wR_2 = 0.0949
Largest diff. peak and hole	0.37/-0.17

Table S18. Selected bond distances (Å) for **BFAC10**.

Atom 1	**Atom 2**	**Bond distances (Å)**
C(1)	O(1)	1.2343(9)
C(1)	N(1)	1.3311(9)
C(2)	N(1)	1.4568(8)

Table S19. Selected bond angles (deg) for **BFAC10.**

Atom 1	Atom 2	Atom 3	Angle (°)
O(1)	C(1)	N(1)	124.56(6)
N(1)	C(2)	C(3)	112.22(6)
C(1)	N(1)	C(2)	120.56(6)

Table S20. Crystal data and structure refinement for **BFAC12**.

Empirical formula	$C_{14}H_{28}N_2O_2$
Formula weight	256.38
Crystal system	triclinic
Space group	P-1
a/Å	4.6030(5)
b/Å	7.5025(8)
c/Å	10.9348(12)
α/°	106.479(4)
β/°	93.005(4)
γ/°	92.023(5)
Volume/Å^3	361.12(7)
Z	1
Calculated density	1.179
F(000)	142.0
Radiation	MoKα (λ = 0.71073)
2 theta range for data collection	5.67 to 55.982
Limiting indices	$-6 \le h \le 6$, $-9 \le k \le 9$, $-14 \le l \le 14$
Reflections collected	12319
Independent reflections	1708 [R_{int} = 0.0462, R_{sigma} = 0.0393]
Data/restraints/parameters	1708/0/86
Quality-of-fit indicator	1.061
Final *R* indices [I>2σ(I)]	R_1 = 0.0421, wR_2 = 0.1123
R indices (all data)	R_1 = 0.0593, wR_2 = 0.1254
Largest diff. peak and hole	0.30/-0.19

Table S21. Crystal data and structure refinement for **BFAC12**.

Empirical formula	$C_{14}H_{28}N_2O_2$
Formula weight	256.38
Crystal system	triclinic
Space group	P-1
a/Å	4.6030(5)
b/Å	7.5025(8)
c/Å	10.9348(12)
α/°	106.479(4)
β/°	93.005(4)
γ/°	92.023(5)
Volume/Å^3	361.12(7)
Z	1
Calculated density	1.179
F(000)	142.0
Radiation	MoKα (λ = 0.71073)
2 theta range for data collection	5.67 to 55.982
Limiting indices	$-6 \leq h \leq 6$, $-9 \leq k \leq 9$, $-14 \leq l \leq 14$
Reflections collected	12319
Independent reflections	1708 [R_{int} = 0.0462, R_{sigma} = 0.0393]
Data/restraints/parameters	1708/0/86
Quality-of-fit indicator	1.061
Final *R* indices [I>2σ(I)]	R_1 = 0.0421, wR_2 = 0.1123
R indices (all data)	R_1 = 0.0593, wR_2 = 0.1254
Largest diff. peak and hole	0.30/-0.19

Table S22. Selected bond distances (Å) for **BFAC12**.

Atom 1	Atom 2	Bond distances (Å)
C(1)	O(1)	1.2308(16)
C(1)	N(1)	1.3372(15)
C(2)	N(1)	1.4623(16)

Table S23. Selected bond angles (deg) for **BFAC12**.

Atom 1	Atom 2	Atom 3	Angle (º)
O(1)	C(1)	N(1)	124.23(12)
N(1)	C(2)	C(3)	112.44(10)
C(1)	N(1)	C(2)	120.48(11)

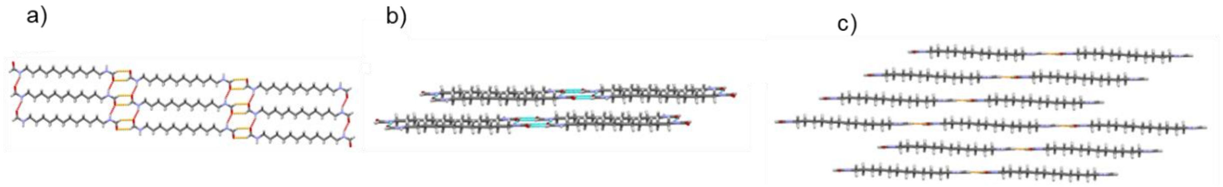

Figure S19. a) Intermolecular hydrogen bonds (N-H···O in red and C-H···O in yellow) of **BFAC12**, b) and c) layered structures of **BFAC12**.

Table S24. Crystallographic data of the investigated compounds

Compound	Space group	a (Å)	b (Å)	c (Å)	α (°)	β (°)	γ (°)
**BFAC4**	$P2_1/n$	5.6495(6)	4.6887(5)	13.6473(15)	90	96.620(4)	90
**BFAC6**	$P2_1/n$	8.1013(5)	4.6433(2)	12.2119(7)	90	95.459(2)	90
**BFAC7**	C2/c	14.0351(5)	4.5728(2)	16.5054(6)	90	110.0330(10)	90
**BFAC8**	$P2_1/c$	7.8234(3)	4.6838(2)	15.1162(6)	90	96.739(2)	90
**BFAC10**	P-1	4.6712(2)	5.4857(2)	12.4311(5)	82.6190(10)	86.0510(10)	84.7860(10)
**BFAC12**	P-1	4.6030(5)	7.5025(8)	10.9348(12)	106.479(4)	93.005(4)	92.023(5)

Table S25. Hydrogen bonds for selected compounds.

Compounds	D	H	A	d(D-H)/Å	d(H-A)/Å	d(D-A)/Å	D-H-A/°
**BFAC2**	N(1)	H(1N)	O(1)[1]	0.896(14)	2.012(15)	2.8633(7)	158.2(12)
**BFAC4**	N(1)	H(1N)	O(1)[2]	0.848(19)	2.047(19)	2.8679(14)	162.7(15)
**BFAC6**	N(1)	H(1N)	O(1)[3]	0.875(17)	2.030(17)	2.8678(13)	160.1(14)
**BFAC7**	N(1)	H(1N)	O(1)[3]	0.862(14)	2.054(13)	2.8593(9)	155.2(11)
**BFAC8**	N(1)	H(1N)	O(1)[3]	0.873(13)	2.026(13)	2.8689(9)	162.0(11)
**BFAC10**	N(1)	H(1N)	O(1)[5]	0.889(11)	2.043(11)	2.8877(8)	158.3(9)
**BFAC12**	N(1)	H(1N)	O(1)[5]	0.925(17)	2.016(19)	2.8895(15)	156.8(15)

Symmetry transformations used to generate equivalent atoms:
[1]1/2-X,1-Y,1/2+Z, [2]1-X,1-Y,1-Z, [3]+X,1+Y,+Z, [4]-1/2+X,1/2-Y,-1/2+Z, [5]-1+X,+Y,+Z, [6]1/2-X,-1/2+Y,3/2-Z, [7]1+X,+Y,+Z

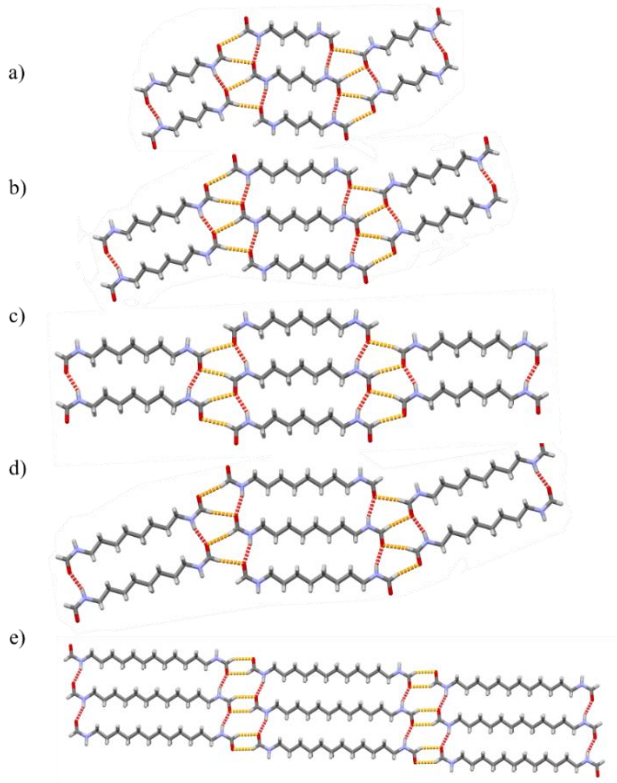

Figure S20. Intermolecular hydrogen bonds (N-H···O in red and C-H···O in yellow) in **BFAC4** (a), **BFAC6** (b), **BFAC7** (c), **BFAC8** (d), **BFAC12** (e).

Table S26. The interlayer distance of **BFACn**.

Compound	Interlayer Distance (Å)
**BFAC4**	3.225
**BFAC6**	3.355
**BFAC7**	3.430
**BFAC8**	3.419
**BFAC12**	3.542

Table S27. The phosphorescence lifetime of compounds investigated.

Compound	**BFAC6**	**BFAC7**	**BFAC8**	**BFAC12**
Average Lifetime (ms)	405	162	138	102

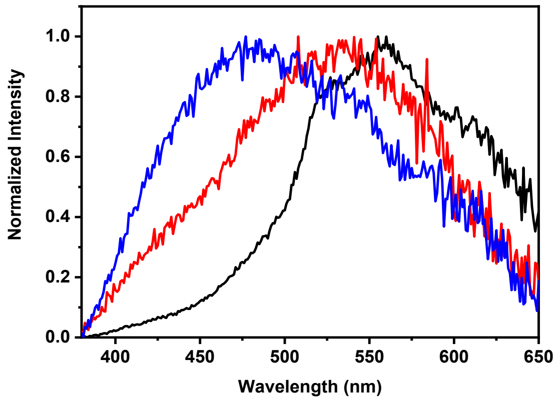

Figure S21. The delayed emission spectra of **BFAC2 (black line)**, **BFAC6 (red line)** and **BFAC12 (blue line)** at 370 nm excitation.

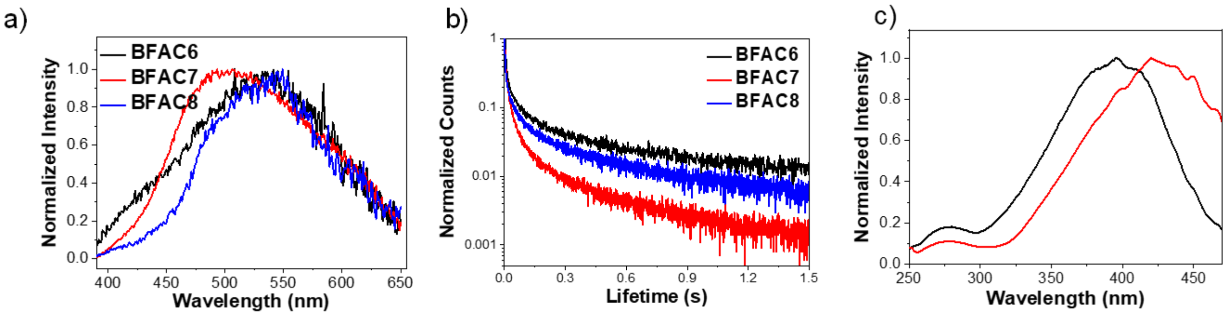

Figure S22. a) Delayed emission spectra of **BFAC6**, **BFAC7** and **BFAC8**, b) luminescence decay plots of **BFAC6**, **BFAC7** and **BFAC8**, c) Excitation spectra of **BFAC6** and **BFAC7** with emission at 500 nm.

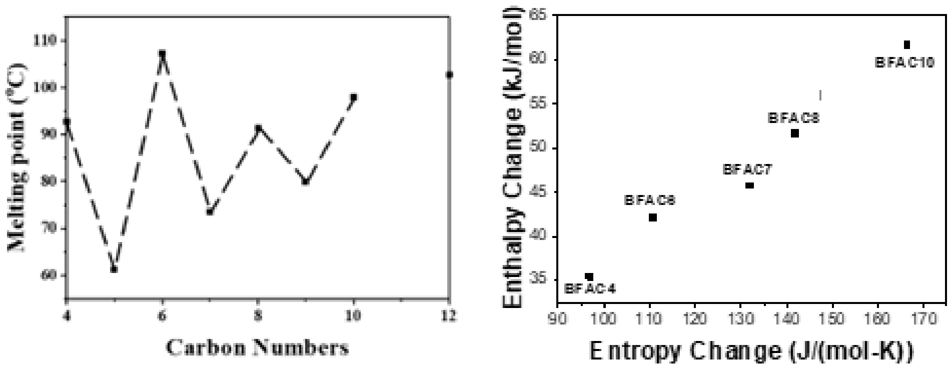

Figure S23. Left: Melting point of **BFACn** compounds; Right: Enthalpy/entropy diagram for BFACn compounds.

References

S1. J. D. Chaney, C. R. Goss, K. Folting, B. D. Santarsiero, M. D. Hollingsworth, "Formyl C−H···O Hydrogen Bonding in Crystalline Bis-Formamides?", *J. Am. Chem. Soc.*, **1996**, *118*, 9432

S2. A. D. Fernando Pulle, H. Frisch, B. T. Tuten, "Direct chromophore integration into polymer backbones via rhodanine step-growth chemistry", *Polym. Chem.* **2025**, 16, 4215.

S3. A. C. Boukis, M. A. R. Meier, "Data storage in sequence-defined macromolecules via multicomponent reactions", *Eur. Polym. J.* **2018**, 104, 32.
